# Distinct Internalization, Acoustic Behavior and Stress Responses to Nanobubbles in Pancreatic Cancer Cells

**DOI:** 10.7150/ntno.130552

**Published:** 2026-09-07

**Authors:** Sila Appak-Baskoy, Muhammad Saad Khan, Dhruvi Patel, Monika Lodyga, Agata A. Exner, Michael C. Kolios, Imogen R. Coe

**Affiliations:** 1 Department of Chemistry and Biology, Toronto Metropolitan University, Toronto, ON M5B 2K3, Canada.; 2 Institute for Biomedical Engineering, Science and Technology (iBEST), Li Ka Shing Knowledge Institute, St. Michael's Hospital, Toronto, ON M5B 1T8, Canada.; 3 Department of Physics, Toronto Metropolitan University, Toronto, ON M5B 2K3, Canada.; 4 Keenan Research Center for Biomedical Science, St. Michael's Hospital, Unity Health Toronto, Toronto, ON M5B 1T8, Canada.; 5 Departments of Radiology and Biomedical Engineering, Case Western Reserve University School of Medicine, Cleveland, Ohio 44106, United States.

**Keywords:** PDAC, nanobubble, * KRAS*, macropinocytosis, ultrasound

## Abstract

Pancreatic ductal adenocarcinoma (PDAC) is one of the most lethal cancers, characterized by an extremely low survival rate once diagnosed. Over 90% of cases harbor activating *KRAS* mutations, which are associated with metabolic reprogramming and altered endocytic activity. New treatment modalities are urgently needed. Phospholipid-shelled, gas-filled nanobubbles (NBs) are promising ultrasound (US) contrast agents and drug delivery vehicles, with potential applications in pancreatic cancer. However, their mechanisms of cellular internalization and the resulting biological responses need to be better understood before they can be effectively utilized as theranostic agents. Here, we analyzed the internalization kinetics, endocytic pathways, acoustic responses, and cell-stress-related protein expression in perfluorocarbon-filled NBs in two pancreatic cancer cell lines: KRAS-mutant PANC-1 and KRAS wild-type BxPC-3 cells showed distinct NB uptake profiles. Flow cytometry demonstrated approximately 1.5-fold higher TR-NB uptake in PANC-1 cells compared with BxPC-3 cells, suggesting enhanced cellular association and/or intracellular retention. Higher NB signal in PANC-1 cells was accompanied by weaker and less punctate LysoTracker staining, whereas BxPC-3 cells displayed brighter, more granular acidic vesicles, suggesting differences in intracellular trafficking and compartmentalization. Pharmacological inhibition of endocytosis revealed that clathrin-mediated endocytosis contributes predominantly to uptake in BxPC-3, whereas macropinocytosis appears to play a greater role in PANC-1 cells. Upon US exposure, NBs exhibited strong initial acoustic activity that declined over time. Analysis of stress-response-associated protein expression revealed cell-specific differences: PANC-1 cells exhibited higher levels of FABP-1, HSP60, SOD2, Thioredoxin-1, and SIRT2, whereas BxPC-3 cells displayed increased COX-2 and PON-2. Collectively, our findings demonstrate that NB internalization and fate differ between PDAC subtypes, potentially reflecting cell type dependent variations in endocytic, lysosomal and adaptive pathways, underscoring the importance of developing cell-tailored nanocarrier designs to enhance delivery precision and therapeutic efficacy.

## Introduction

Targeted therapies for cancer have advanced considerably over the past two decades, evolving from receptor-specific monoclonal antibodies and kinase inhibitors to highly engineered delivery platforms capable of controlling the location, timing, and intensity of therapeutic release 1. Despite these advances, the clinical impact of targeted therapeutics remains limited by several barriers, including heterogeneous target expression within tumors, dense stromal tissue, and systemic toxicities resulting from non-specific distribution of drugs 2. These limitations underscore the need for delivery systems that combine stability in systemic circulation with externally controllable, site-specific release. Ultrasound (US)-responsive carriers are an emerging solution to this problem, enabling spatiotemporal control of therapeutic release, thereby maximizing efficacy at the tumor site while reducing systemic exposure 3.

Among acoustically responsive carriers, microbubbles (MBs) and nanobubbles (NBs) have attracted substantial interest. MBs, typically 1-10 µm in diameter, have been widely used as contrast agents in clinical US imaging due to their strong echogenicity. However, their relatively large size confines them to the intravascular compartment, limiting their potential for extravasation into tumor tissue and preventing direct intracellular delivery 4,5. In contrast, NBs with a mean diameter of approximately 250 nm are small enough to circulate through capillary beds and extravasate into tumor interstitium via the enhanced permeability and retention effect. This fundamental distinction makes NBs a more versatile platform, serving as both diagnostic enhancers and therapeutic delivery vehicles at the cellular and subcellular levels 6,7,8,9. Structurally, NBs consist of a stabilizing shell, composed of lipids, polymers, surfactants, or proteins, surrounding a hydrophobic gas core such as octafluoropropane or sulfur hexafluoride. The shell exhibits viscoelastic behavior, enabling linear and nonlinear oscillations when exposed to US 10,11. These oscillations drive acoustic cavitation, which can temporarily increase membrane permeability, enhance drug penetration, and, under certain conditions, directly induce apoptosis in tumor cells 9,12. Their biocompatibility, tunable surface chemistry, circulation stability, and potential for functionalization with targeting ligands further strengthen their potential as precision delivery platforms 3,13.

The relevance of such systems is particularly high for malignancies driven by *KRAS* mutations, which remain one of the most intractable molecular targets in oncology. KRAS mutations are highly prevalent in solid tumors, occurring in 40-45% of colorectal cancers, 25-30% of non-small cell lung cancers, and more than 90% of pancreatic ductal adenocarcinoma (PDAC) cases 14,15. PDAC is particularly challenging due to late diagnosis, poor responsiveness to chemotherapy, and a five-year survival rate that remains very low 16,17. *KRAS*-driven cancers are characterized by altered intracellular trafficking and activation of multiple downstream signaling pathways, including RAF/MEK/ERK and PI3K/AKT/mTOR cascades, as well as non-canonical nutrient-scavenging processes such as macropinocytosis and autophagy 18,19 which not only drive therapy resistance but may also reshape how the tumor cells interact with nanoscale delivery systems.

Despite the increasing interest in NBs as contrast agents, the mechanisms by which they are internalized into cells remain poorly understood, particularly in *KRAS*-associated tumors, where intracellular trafficking and nutrient-scavenging pathways are known to be altered therefore we aimed to characterize NB internalization pathways in pancreatic cancer cells.

Nanoparticles frequently exploit clathrin-mediated endocytosis (CME) as an uptake pathway 20, while *KRAS*-mutant cancer cells demonstrate enhanced macropinocytosis, a scavenging process by which extracellular fluids and nutrients are internalized 20,19,21. While targeted NBs, such as prostate-specific membrane antigen (PSMA)-functionalized formulations, have been shown to enter prostate cancer cells through CME 8, the uptake mechanisms of non-targeted NBs in PDAC cells remain poorly defined. In particular, it is unclear how NB uptake is partitioned between CME and macropinocytosis, how this varies across PDAC cell types, and whether NBs retain acoustic responsiveness following cellular interaction and intracellular trafficking which are important for optimizing NB-based theranostic strategies requiring efficient uptake and on-demand ultrasound activation. Addressing these questions is important for optimizing NB-based theranostic strategies that require efficient cellular uptake and controlled US activation.

Finally, although combined MB-US treatment has been associated with oxidative stress in cancer cells 22, it remains unclear whether NBs, which can also be used as drug carriers contribute to oxidative stress. Therefore, this study seeks to investigate the differences between BxPC-3 cells and PANC-1 cells in NB uptake, mechanisms of NB internalization, acoustic responsiveness, and to determine whether NB internalization induces cellular stress responses. By clarifying these fundamental aspects of NB biology, this work aims to guide the rational design of NB systems that maximize therapeutic precision, enhance on-target efficacy, and minimize off-target and toxic effects.

## Materials and Methods

### 1. NB Preparation and Characterization

TexasRed-labeled (TR) NBs and unlabelled PGG were prepared as described previously 23. Briefly, 1 mg of Texas Red (TR)® DHPE (Biotium) was dissolved in chloroform (1 mg/mL). After evaporation, lipids: DBPC (60.1 mg), DPPE (20 mg), DPPA (10 mg), and DSPE-mPEG2k (10 mg) were added. The same lipid ratios, except TR, were used to make the unlabelled NBs. After the addition of Propylene glycol (1 mL), the mixture was heated at 80 °C and sonicated until dissolved. A pre-heated solution of glycerol (1 mL) and PBS (8 mL) was added, followed by sonication at room temperature for 10 min. The final 10 mL lipid solution was aliquoted and stored at 4 °C for up to 2 weeks.

For activation, 1 mL lipid solution was added to a 3 mL vial, which was sealed, and air was manually removed using a 25 mL syringe. Subsequently, the headspace was filled with octafluoropropane (C₃F₈) gas. The vial was then shaken for 45 sec using a VialMix (Bristol-Myers Squibb Medical Imaging, MA). For NB isolation, the inverted vial was centrifuged (550 rpm, 5 min) to collect NBs at the bottom of the vial. A modified 25G needle was used to extract 400 µL of NB solution.

NBs were analyzed using the Archimedes resonant mass spectrometry system (Malvern Panalytical). Samples were diluted 1:1000 in PBS, and 1000 particles were counted in each sample. Positively buoyant particles have been reported in size measurements. For the US and OD experiments, PGG NBs were used, which have the same lipid and gas composition but lack the fluorescent TR component.

### 2. Cell Culture

PDAC cell lines PANC-1 and BxPC-3 were used for the experiments. PANC-1 cells (ATCC CRL-1469) were cultured in Dulbecco's Modified Eagle's Medium (DMEM; Gibco™, Thermo Fisher Scientific, Milano, cat# LS11965092) supplemented with 10% (v/v) fetal calf serum (FCS; Gibco™, Thermo Fisher Scientific, Milano, cat# 10437-036). The cells were maintained at 37 °C in a humidified atmosphere with 5% CO₂ (v/v). Subculturing was performed at a 1:5 ratio using 0.025% (v/v). Trypsin-EDTA solution (Thermo Fisher Scientific, cat# 15090046). BxPC-3 cells (ATCC CRL-1687) were cultured in Roswell Park Memorial Institute (RPMI) 1640 medium (Gibco™, Thermo Fisher Scientific, Milano, cat# 11875093) supplemented with 10% (v/v) fetal calf serum (FCS; Gibco™, Thermo Fisher Scientific, Milano, cat# 10437-036). The cells were maintained at 37 °C in a humidified atmosphere with 5% CO₂. Subculturing was performed at a 1:5 ratio using 0.025% Trypsin-EDTA solution (Thermo Fisher Scientific, cat# 15090046).

### 3. Flow Cytometry Analysis of NB Internalization

PANC-1 and BxPC-3 cells were harvested using trypsin, and 500,000 cells were resuspended in 500 µL of HBSS (Stemcell Technologies, #37150). PANC-1 cells are adherent; however, under the culture conditions they tended to form multilayered aggregates, and superficial cells were more susceptible to loss during washing/fixation, therefore TR-NBs incubations were carried out when cells were in suspension in 2ml tubes. TR-NBs were added at varying concentrations corresponding to 2 × 10⁴, 1 × 10⁴, and 5 × 10³ nanobubbles (NBs) per cell. Cells were incubated with TR-NBs at three temperatures (4 °C, 37 °C, and 42 °C) and collected at four time points (1, 30, 60, and 90 minutes). Following incubation, cells were immediately placed on ice, centrifuged at 200 × g for 5 minutes, and gently washed with cold flow buffer (PBS containing 1% BSA). After a final centrifugation step, cells were resuspended in 250 µL of flow buffer supplemented with DAPI (as a viability dye) immediately prior to acquisition. Samples were acquired using a CytoFLEX-LX (Beckman Coulter) flow cytometer operated with CytExpert v2.6 software. Texas Red fluorescence was excited at 561 nm and detected using a 610/20 nm bandpass filter, while DAPI was excited with a 405 nm laser and detected using a 450/45 nm bandpass filter. A minimum of 8,000 single, live (DAPI-negative) events were collected per sample and analyzed using FlowJo (BD) software. Internalization of TR-NBs was assessed in single, viable cells (see Supplementary Figure 1a for gating strategy). The TR-positive gate was defined based on the DAPI single-stain control. Data for the TR-positive population were expressed as both the frequency of positive events and the mean fluorescence intensity (MFI) of the Texas Red signal.

For both internalization frequency and MFI, data were normalized to each own 4 °C condition time point (at which endocytic internalization is very slow), for example, the 37 °C, 30-minute condition was normalized to the 4 °C, 30-minute control) (see Supplementary Figure 1b for schematic). This normalization ensures that the reported increases in TR-NB uptake at the various temperature and time points reflect intracellular uptake rather than surface binding.

### 4. Microscopy

PANC-1 or BxPC-3 cells (200,000) were seeded in 35 mm glass-bottom dishes with 1.5 mm coverslips (Cellvis, # D35-14-1.5N) and allowed to adhere overnight. Cells were divided into 1, 30, and 60 minute incubation groups. To accurately determine the number of NBs added per cell, cells from two additional dishes were counted to calculate the average cell number. Cells were incubated with TR-NBs in 1 ml HBSS at 37 °C for the indicated times. After incubation, cells were fixed with 4% paraformaldehyde for 10 minutes at room temperature and washed with PBS. Membrane staining was carried out with Wheat Germ Agglutinin conjugated to Alexa Fluor 488 (Thermo Fisher, #W11261) and nuclear staining was done with DAPI (Thermo Fisher, #D1306). Images were acquired using a Zeiss Wide Field fluorescence microscope. For the lysotracker staining, cells were incubated with TR-NBs for 1 hour, washed and then cells were incubated with LysoTracker Green DND-26 (Thermo Fisher Scientific, #L7526) for 20 min at 37°C to label acidic intracellular compartments, cells were then washed and fixed with 4% PFA. For the lysotracker imaging, a spinning disk confocal microscope, Quorum Spinning Disc Microscope equipped with an EM-CCD camera was used and images were acquired using a 63×/1.4 NA oil immersion objective.

### 5. Pharmacological Inhibition of Endocytosis Pathways

BxPC-3 and PANC-1 cells (800,000) were seeded in wells of 6 well plates in 2 mL of RPMI-1640 (for BxPC-3) or DMEM (for PANC-1) media. Cells were pre-treated for 20 minutes with either 1.5 µM or 4 µM Ikarugamycin (IKA) for BxPC-3 and PANC-1 cells, respectively (Cayman Chemical, #36531-78-9), or with 50 µM EIPA for both cell lines (Cayman #1154-25-2). For CME inhibition, we initially used 4 μM IKA (Elkin *et al*., 2016), but this concentration caused > 80% cell death in BxPC-3 cells. To identify a non-toxic yet effective dose, we tested lower concentrations and found that 1.5 μM IKA maintained cell viability (>80%) while significantly reducing transferrin internalization (Supplemental Figure 2). Following the pre-treatment with the inhibitors, a total of 1 × 10⁴ TR-NBs were added per cell and incubated with cells at 37 °C for 60 minutes. After the incubation, dishes were immediately placed on ice and cells were gently washed with 2 mL of 1x PBS to remove unbound NBs. Viability was assessed by flow cytometry using eFluor780 (e780) fixable viability dye (Thermo Fisher# 65-0865-14). e780 was selected because EIPA exhibits intrinsic fluorescence near 400 nm, which interferes with DAPI detection, and NBs were labeled with Texas Red (~615 nm); thus, the far-red e780 channel avoids spectral overlap with both.

### 6. US Exposure

A water tank with a 3D positioning system was used to conduct the US experiments. A 3D positioning system was used to hold the treatment transducer, sample holder and PCD transducer. The treatment transducer (PZT, 1 MHz, Precision Acoustic UK) and the PCD transducer (PVDF, 3-10 MHz bandwidth, Precision Acoustics UK) were orthogonal to each other. The treatment transducer was operated at a peak negative pressure (PNP) of 770 kPa, 1% duty cycle, 500 Hz PRF, and a transmit frequency of 1 MHz for 1 min. Pulses were generated using a waveform generator (Agilent 33521A, Agilent Technologies, CA, USA) and further amplified using an RF amplifier (BSD Medical, USA). Signal acquisition and processing details for our PCD system are reported elsewhere 25.

US treatments were conducted using PGG-coated NBs (PGG-NBs), as detailed in Supplemental Table 1, with an average diameter of 256 nm and 89% positive buoyancy. Four experimental groups were prepared. NB-only control: PGG-NBs (10,000 NBs per cell) were incubated in 2 ml of HBSS at 37 °C for 1, 30, or 60 minutes, then exposed to US to measure baseline NB acoustic signal. Cell-only control: 2.1 × 10⁶ cells were incubated in 2 ml of HBSS at 37 °C for the same time points, followed by US to measure the background acoustic signal from cells alone. NB-treated cells: 2.1 × 10⁶ cells were incubated with PGG-NBs (10,000 per cell) in 2 ml of HBSS at 37 °C for 1, 30, or 60 minutes. Cells only: Cells incubated at 37 °C for the same time points without NB or US exposure for viability control. After incubation, all samples were immediately placed on ice, and US treatment was performed. For all US treatments, the sample volume was adjusted to 4 ml with HBSS. Samples were transferred to a cylindrical sample container constructed from Mylar film, which was attached to a 3D positioning system. A magnetic stirrer was placed inside the container during US exposure to prevent cell sedimentation and NB aggregation.

Moku:lab (Liquid Instruments, CA, USA) was used to acquire and record signals using a MATLAB API. The received PCD signal was amplified using a 30 dB preamplifier (LNA 150, RF Bay, Inc., MD, USA). Acoustic absorbers (Aptflex F28P, Precision Acoustics, UK) were added to the water tank to reduce noise and absorb unwanted scattering. Postprocessing was done in MATLAB to acquire power spectra using Pwelch method. Power spectra were normalized to the power spectra obtained from radiofrequency (RF) signals received from cell controls, and the integrated power spectra were calculated by integrating the normalized signal over the frequency range of 0.2 to 10 MHz. Four independent biological replicates were performed, and two samples per condition were acquired in each experiment. The plotted curves represent the averaged signals across replicates, and a moving average (n = 16) was applied for smoothing.

### 7. Optical Density (OD) Measurements

OD measurements were conducted before and after the US treatment. Optical density was measured using a custom-developed system. A 1 mL aliquot of the sample was pipetted into a cuvette and mounted in a cuvette holder (CVH100, Thorlabs, USA). Broadband halogen illumination was delivered to the cuvette holder via an optical fiber, attenuated by the sample, and the transmitted light was collected with a second optical fiber and directed to a spectrophotometer (USB4000, Ocean Optics, USA). Absorbance was determined by comparing the transmitted intensity to a reference using a logarithmic ratio, and OD600 was reported as the absorbance at 600 nm.

### 8. Protein Isolation and Quantification

A total of 1 x 10⁶ cells were incubated with 1 x 10^4^ PGG-NBs per cell for 1 hour at 37 °C. Following treatment, cells were lysed using the lysis buffer provided in the R&D Systems Human Stress Response Proteome Profiler Array kit (R and D #: ARY018), according to the manufacturer's instructions. Protein concentration was determined using the Pierce™ BCA Protein Assay Kit (Thermo Scientific #: 23227), following the manufacturer's instructions. Briefly, working reagent (WR) was prepared by mixing Reagent A and Reagent B at a 50:1 ratio. Samples and bovine serum albumin standards (0-2000 µg/mL) were loaded into a 96-well plate, and 25 µL of each sample or standard was added per well in duplicate. Subsequently, 200 µL of WR was added to each well, mixed thoroughly, and the plate was incubated at 37 °C for 30 minutes. Absorbance was measured at 562 nm using a microplate reader. Protein concentrations were calculated based on the BSA standard curve and 150 µg of protein was used for the array.

### 9. Profiling of Cell Stress Protein Expression

Cell stress protein profiling was conducted using the Proteome Profiler Human Cell Stress Array Kit (R&D Systems, ARY018) according to the manufacturer's instructions. A total of 150 μg of protein from cell lysates was incubated with a cocktail of biotinylated detection antibodies and applied to nitrocellulose membranes pre-spotted with capture antibodies for 26 human stress-related proteins. After overnight incubation at 4 °C, membranes were washed, incubated with streptavidin-HRP, and developed using a chemiluminescent substrate. Images were acquired using the Bio-Rad ChemiDoc imaging system, and signal intensities were quantified using Quick Spots image analysis software. Duplicate spots were averaged, and background-subtracted signals were compared between untreated and NB-treated groups.

### 10. Statistics

Three independent experiments were conducted for the statistical analysis, which were performed using GraphPad Prism version 8.0 for Windows (GraphPad Software, San Diego, CA, 2018). Mean ± SD was shown for all data. A 2-way analysis of variance (ANOVA) was conducted to assess group differences, with Tukey's or Sidak's HSD test for intra-group comparisons. (*) P < 0.05, (**) P < 0.01, (***) P < 0.001, (****) p < 0.0001.

## Results

### NB Characterization and Selected NB to Cell Ratio

To determine the nature of cellular internalization of NBs, we used octafluoropropane-cored phospholipid Texas Red (TR)-labelled NBs, which were characterized using Archimedes resonant mass measurement (ARMM) (Malvern Panalytical, Malvern, Worcestershire, UK). ARMM analysis indicated that TR-NBs used across experiments (17 independent preparations) had an average diameter of approximately 290 nm, with over 86% exhibiting positive buoyancy and an average concentration of 4.7 × 10⁸ particles/mL, confirming the predominance of gas-filled particles. (Supplemental Table 2).

To determine the number of NBs, we exposed cells to 5 × 10³, 1 × 10⁴, and 2 × 10⁴ NBs per cell for 1 hr, and evaluated internalization by flow cytometry. Since cellular uptake of nanomaterials is typically temperature-dependent, we hypothesized that TR-NB internalization would increase with temperature. Therefore, we compared uptake at 4 °C, 37 °C, and 42 °C. As predicted, at 4°C, there was minimal internalization, with only surface binding, consistent with previous findings (Nagai *et al*., 2019) (Figure 1 a,b) and we used the 4 °C condition as a baseline control to account for background fluorescence. In contrast, at 37 °C, we observed a marked increase in cellular fluorescence in both BxPC-3 and PANC-1 cells, confirming temperature-dependent internalization of TR-NBs (Figure 1a, b). As expected, the highest internalization occurred at 42 °C. In addition, PANC-1 cells consistently exhibited greater internalization than BxPC-3 cells across all NB numbers and temperatures (Figure 1 a,b). Among the tested conditions, exposure to 1 × 10⁴ NBs per cell produced the highest median fluorescence intensity (MFI), comparable to or slightly lower than that observed with 2× 10⁴ NBs, but significantly higher than the condition with 5 × 10³ NBs across temperatures (Figure 1a,b). 1 × 10⁴ TR-NBs per cell represented an ideal condition, balancing material use and internalization efficacy, and was therefore selected for subsequent experiments.

### Time Course Analysis of Internalization

We next examined the kinetics of TR-NB uptake under varying incubation times and temperatures to determine how these parameters influence cellular association/internalization. At 4°C, both BxPC-3 and PANC-1 cells showed minimal uptake across all time points, consistent with limited energy-dependent internalization under low-temperature conditions (Figure 2a-d).

In BxPC-3 cells, the percentage of NB-positive cells increased over time at 37°C and 42°C, reaching the highest values at 60-90 min (Figure 2c,g). However, normalized MFI remained relatively stable, with only modest increases that became significant primarily at later time points (Figure 2e,h) suggesting that prolonged incubation increased the number of BxPC-3 cells associated with TR-NBs, while the average fluorescence signal per positive cell changed only modestly.

In contrast, PANC-1 cells exhibited a more pronounced time and temperature dependent response. The proportion of NB-positive cells increased progressively from 30 to 90 min, with the strongest uptake observed under mild hyperthermic conditions (42°C) (Figure 2d,g). In parallel, MFI also increased significantly at 60 and 90 min, particularly at 42°C (Figure 2f,h). Compared with BxPC-3 cells, PANC-1 cells therefore demonstrated both a higher fraction of TR-NB-positive cells and greater fluorescence intensity per cell at later time points, indicating enhanced TR-NB cellular association and/or intracellular accumulation.

Notably, across both cell lines, changes in the frequency of NB-positive cells were generally more pronounced than changes in MFI, suggesting that hyperthermia primarily increased the proportion of cells interacting with TR-NBs rather than dramatically increasing the per-cell NB burden. Mild hyperthermia therefore enhanced TR-NB cellular association overall, with the greatest effect observed in PANC-1 cells. The enhanced response in PANC-1 cells may be related to intrinsic differences in membrane trafficking or endocytic activity, consistent with prior reports describing elevated uptake pathways in KRAS-driven pancreatic cancer cells 19.

### Temporal Analysis of Fixed Cells by Fluorescence Microscopy

We characterized the intracellular trafficking of TR-labeled nanobubbles (TR-NBs) by examining their spatiotemporal distribution in fixed cells using wide-field fluorescence microscopy. In agreement with the flow cytometry findings, incubation at 4 °C for 60 minutes resulted in negligible TR-NB fluorescence, confirming that nanobubble internalization is effectively halted at low temperature (Figure 3a,b), supporting the concept that NB internalization requires adequate membrane fluidity and metabolic energy, consistent with the temperature-dependent phase behavior of lipid bilayers 27.

At 1 minute post-exposure (37 °C), TR-NBs were predominantly localized at the cell membrane, representing rapid initial surface association (Figure 3c,d). By 30 minutes, TR-NBs were internalized and distributed throughout the cytoplasm, suggesting active endocytic uptake and early intracellular trafficking. At 60 minutes, TR-NBs accumulated in the perinuclear region, implying progression into late endosomal or perinuclear vesicular compartments (Figure 3c,d) confirming a time and temperature dependent redistribution of TR-NB derived fluorescence from the membrane to the perinuclear region, consistent with active internalization and intracellular trafficking. However, it is also possible that the observed signal at later stages reflects lipid shell remnants rather than intact gas-filled NBs. Furthermore, a clear difference in lysosomal acidification was observed between the two cell lines. BxPC-3 cells exhibited strong lysosomal acidification, whereas PANC-1 cells showed markedly weaker Lysotracker staining (Figure 3e,f). After 60 minutes of exposure, TR-NBs were largely colocalized with acidified lysosomes in BxPC-3 cells (Figure 3e). In contrast, PANC-1 cells showed weaker Lysotracker staining suggesting NB localization in less acidic endosomal compartments or the Golgi rather than mature lysosomes (Figure 3f).

### Mechanism of Internalization

As nanoparticles are mainly internalized via CME or macropinocytosis, we next examined which pathway governs TR-NB internalization by selectively inhibiting CME and macropinocytosis using IKA and EIPA, respectively. The efficacy of 50 μM EIPA was confirmed using 70 kDa fluorescent dextran, which verified effective blockage of macropinocytosis in both PANC-1 and BxPC-3 cells, as indicated by the reduced fluorescent dextran uptake in both cell lines (Supplemental Figure 3).

Flow cytometric analysis revealed uptake patterns between the two cell lines (Figure 4a, b). In BxPC-3, IKA reduced the proportion of TR-NB-positive cells by 41%, whereas EIPA reduced it by only 7%, indicating a predominant role for CME in these cells (Figure 4c). In contrast, PANC-1 cells exhibited a stronger dependence on macropinocytosis than CME: EIPA reduced TR-NB-positive cells by 69.2%, compared with 49.6% following CME inhibition (Figure 4e). Across all treatments, cell viability was above 80% (Supplemental Figure 4).

Moreover, MFI, representing the fluorescence signal measured per cell, also showed differences. CME inhibition with IKA reduced the mean fluorescence intensity (MFI) by 55% in BxPC-3 cells, whereas macropinocytosis inhibition with EIPA decreased it by 30% (Figure 4d). In PANC-1 cells, EIPA treatment reduced MFI by 58%, compared to a 48% reduction with IKA, confirming that individual PANC-1 cells can activate both uptake mechanisms to a similar extent, with a greater reliance on macropinocytosis (Figure 4f). In contrast, CME appears to be the primary internalization route in BxPC-3 cells. Together, these data demonstrate that TR-NBs are internalized via both CME and macropinocytosis, with cell-type-specific preferences reflecting divergent endocytic programs in pancreatic cancer subtypes, prompting further investigation into the US response of the NBs within cells.

### Cell Associated US Response of Internalized TR-NBs

After confirming TR-NB internalization and the underlying endocytic mechanisms, we next assessed whether internalized NBs remained acoustically active (responsive to ultrasound) at 1-, 30-, and 60-minutes post-incubation at 37 °C. If internalization and intracellular processing alter NB integrity, then we would expect a progressive decline in acoustic activity over time. As expected, at the 1 minute time point, NBs exhibited strong acoustic signals both in suspension and during cellular incubation, consistent with their membrane-associated localization observed by fluorescence microscopy and the early internalization detected by flow cytometry (Figure 5). By 30 minutes, the acoustic signal was reduced, suggesting that a substantial fraction of NBs had been degraded, internalized, or stabilized in non-acoustically responsive states, thereby diminishing the US PCD signal. After 60 minutes, acoustic activity was minimal and largely stable, indicating that most NBs had either been fully internalized, sequestered into intracellular compartments in non-acoustically responsive states, or had lost gas content required for echogenicity. Time-dependent decline in acoustic response supports the notion that NB-cell interactions and subsequent internalization progressively dampen US activity. Importantly, when we compare 30 min and 60 min lines for PANC-1 and BxPC-3, we observe 1-2 dB higher signal for PANC-1 indicated by black vertical line, but this observation doesn't stay consistent throughout acquisition and therefore it is fair to conclude that both cell lines exhibit similar response. This is consistent with the MFI data of TR-NB internalization at 37 °C for both cell lines that show although the frequency of TR-NB internalization by PANC-1 cells is higher the TR intensity per cell remains similar for both cell lines at 37 °C. Moreover, cell viability remained unchanged between treated and untreated groups (Supplemental Figure 5a,b), confirming that NB exposure and US activation were non-cytotoxic under the tested conditions.

### Time-Dependent Decline in Turbidity

During PGG-NB cell incubation experiments, we observed a progressive decrease in the turbidity of the HBSS over time, which became more apparent after US exposure. HBSS appeared most opaque at the early time point (1 min) and gradually became less turbid after longer incubation periods (30 and 60 min) (Supplementary Figure 6a,b). Following US exposure, a reduction in OD was observed, particularly at the 1 min time point, consistent with disruption of acoustically responsive extracellular NBs. At 30 and 60 min, the US-induced OD decrease was less pronounced, suggesting that fewer suspended NBs remained available to respond to US after prolonged incubation. Because no wash step was performed prior to measurement, the OD values reflect the combined contributions of cells together with extracellular suspended NBs and any cell-associated NBs remaining in the suspension. Therefore, the observed decrease may reflect cellular association and/or uptake of NBs, as well as spontaneous NB loss during incubation. Notably, cell-only controls showed minimal change in OD before and after US treatment, indicating that the observed turbidity changes were primarily attributable to NBs rather than cell disruption or sedimentation (Supplementary Figure 6c,d).

### Cellular Stress Protein Expression upon NB Internalization

We hypothesized that if NB internalization imposes a cellular burden and if macropinocytosis-driven uptake in PANC-1 cells leads to greater NB accumulation, then these cells should exhibit a heightened stress response compared with BxPC-3. We profiled 26 stress- and survival-related proteins in BxPC-3 and PANC-1 cells following 60 minutes of NB exposure using the Proteome Profiler Human Cell Stress Array Kit (Supplemental Figure 7).

Indeed, PANC-1 cells displayed more than 2-fold upregulation of multiple stress-related proteins, including HSP60, fatty acid-binding protein-1 (FABP-1), superoxide dismutase-2 (SOD2), thioredoxin-1 (TRX-1), and the metabolic stress regulator SIRT2, indicating an integrated cellular defense program involving proteostasis maintenance, redox buffering, metabolic adaptation, and oxidative stress mitigation (Figure 6b).

In contrast, BxPC-3 cells exhibited a more modest response, with only a 1.9-fold upregulation of cyclooxygenase-2 (COX-2) and a 1.3-fold increase in paraoxonase-2 (PON-2), enzymes associated with inflammatory signalling and oxidative defence (Figure 6a). Therefore, we show that NB internalization activates distinct stress response networks in pancreatic cancer cells, with PANC-1 cells mounting a broader metabolic and proteostatic adaptation, whereas BxPC-3 cells primarily engage inflammatory and antioxidant pathways that may enable cells to recover from or withstand NB-induced stress, potentially leading to increased resistance to subsequent treatments.

## Discussion

NBs represent a nanoscale evolution of MB technology, expanding the utility of US-mediated imaging and drug delivery beyond vascular confinement. While MBs are limited to the bloodstream due to their micron-scale size, NBs, by virtue of their submicron dimensions, can penetrate leaky tumor vasculature and diffuse into the interstitial space, enabling deeper tissue access and improved therapeutic distribution. Phospholipid shells of NBs further enable chemical modification, such as PEGylation or ligand conjugation, enhancing circulation stability and tissue specificity 28, making NBs powerful theranostic platforms that combine real-time US imaging with precision-targeted therapy. Despite this potential, the mechanisms governing NB internalization, particularly for non-targeted formulations, remain poorly understood. Most prior studies have focused on ligand-functionalized NBs and their CME in select tumor models 8. However, how non-targeted NBs interact with cells and how cell-intrinsic factors influence these interactions are less clear. To address this gap, we investigated the uptake dynamics of perfluorocarbon-filled NBs in two genetically and morphologically distinct BxPC-3 and PANC-1 cells, integrating flow cytometry, imaging, pharmacological inhibition, and molecular analysis. Our results demonstrate that NB internalization is rapid. Within one minute of exposure, NBs accumulated at the plasma membrane, followed by gradual translocation toward the perinuclear region within 60 minutes, which is parallel to those described for other nanoscale systems, such as carboxylated polystyrene nanoparticles (40-200 nm), which undergo transient membrane adsorption prior to internalization 29. Prolonged incubation resulted in increased intracellular levels of TR-NBs, with BxPC-3 cells exhibiting a delayed accumulation that became apparent at later time points (90 min), suggesting that, in BxPC-3 cells, early internalization of TR-NBs is balanced by efficient intracellular processing and/or exocytosis, whereas continued exposure ultimately leads to accumulation. In addition, exposure to elevated temperature (42 °C) further increased TR-NB accumulation in both cell lines. Hyperthermia is known to enhance membrane fluidity and stimulate energy-dependent endocytic pathways, which can increase TR-NB internalization 30. Elevated temperature may also transiently disrupt intracellular trafficking processes, thereby promoting intracellular retention of TR-NBs. *KRAS*-mutant PANC-1 cells exhibiting markedly higher internalization than *KRAS* wild-type BxPC-3 cells, aligns with the enhanced macropinocytic activity driven by oncogenic *KRAS* to sustain metabolic and redox balance 21,31. Pharmacological inhibition of endocytosis confirmed that BxPC-3 cells primarily rely on CME, whereas PANC-1 cells can activate more macropinocytosis than CME for internalization of NBs. Given that clathrin-coated vesicles are restricted to cargos under ~200 nm 32 and NBs often slightly exceed this range, their preferential uptake in PANC-1 cells underscores the role of macropinocytosis in accommodating larger nanoscale entities. This divergence also illustrates how different PDAC cell types remodel endocytic pathways and may offer opportunities to exploit macropinocytosis for genotype-specific nanocontrast delivery. Moreover, simultaneous inhibition of CME and macropinocytosis induced over 90% cell death in both PDAC lines, underscoring the essential roles of these two endocytosis pathways in maintaining metabolic and cellular homeostasis.

In addition, at 4 °C, TR-NBs associate with the cell surface but are not internalized. In contrast, at 37 °C they enter cells, indicating that internalization is temperature-dependent and likely influenced by membrane fluidity, consistent with energy-dependent endocytic mechanisms being inhibited by membrane rigidity at low temperature 27. Furthermore, the reduced Lysotracker signal observed in PANC-1 cells suggests altered lysosomal acidification and spatial organization relative to BxPC-3 cells. The confinement of weakly positive vesicles to discrete perinuclear regions in PANC-1 cells, rather than a continuous perinuclear ring as observed in BxPC-3 cells, indicates spatially restricted lysosomal maturation and pH heterogeneity that may lead to weaker steady-state Lysotracker labeling despite continued reliance on lysosome-mediated processing. The efficient lysosomal acidification observed in BxPC-3 cells may facilitate more rapid TR-NB processing and degradation, leading to delayed accumulation that becomes evident only at later exposure times. Additional characterization using markers of early endosomes (EEA1 or Rab5) and late endosomes/lysosomes (LAMP1) would further substantiate the intracellular trafficking route of TR-NBs.

Interestingly, NBs were internalized even under serum-free conditions, revealing that uptake does not rely on protein corona formation. This points to direct recognition by cell-intrinsic receptors or physicochemical interactions at the membrane interface. Potential mediators include scavenger receptors (SR-B1, CD36, LDLR family), integrins, or curvature-sensitive CME variants driven by electrostatic or mechanical forces 33. Taken together, TR-NB uptake without the need of protein corona is primarily governed by intrinsic NB properties, which is advantageous for robust and predictable tumor imaging applications.

When exposed to US at early incubation time points, optical density (OD600) measurements showed a pronounced decline after treatment, consistent with disruption or loss of suspended NBs present in the bulk medium. This was supported by PCD, where integrated acoustic responses were highest at 1 min and 30 min and progressively decayed during sonication, indicating a larger population of acoustically responsive NBs at these earlier time points. At later incubation times (60 min), the change in OD before and after US exposure was markedly reduced, and OD values of the NB + cell groups approached those of cell-only controls. Likewise, integrated PCD responses at 60 min were substantially lower and, in cell-incubated samples, approached baseline levels. Together, these findings suggest that fewer freely suspended extracellular NBs remained available to contribute to turbidity or generate detectable cavitation signals after prolonged incubation. Time-dependent reduction in extracellular acoustic activity may reflect cellular association and/or internalization of NBs, spontaneous NB dissolution, or altered responsiveness of NBs in the cellular microenvironment. However, because samples were not washed prior to US exposure, the present measurements represent the combined contributions of extracellular, cell-associated, and potentially internalized NBs. Therefore, these data do not independently distinguish intracellular NB activity from residual extracellular NB activity, but are consistent with a progressive decrease in the acoustically active NBs over time.

At the molecular level, NB exposure elicited distinct protein-expression responses, revealing the functional impact of these differential uptake routes. In BxPC-3 cells, NB internalization induced selective upregulation of *COX-2* and *PON-2*, key regulators of inflammation and oxidative defense. *COX-2* mediates prostaglandin biosynthesis and is linked to tumor-associated inflammation 34, whereas *PON-2* protects against lipid peroxidation and oxidative stress 35. This protein-expression pattern suggests that NB internalization triggers a hybrid inflammatory antioxidant response in wild-type cells, likely as a protective adaptation to membrane perturbation. In contrast, PANC-1 cells displayed a broader and more coordinated cytoprotective program. The upregulation of *HSP60*, *FABP-1*, *SOD2*, *Thioredoxin-1*, *SIRT2* indicates activation of an integrated stress response involving proteostasis, redox buffering, and mitochondrial adaptation. Such patterns are consistent with enhanced NRF2-driven antioxidant capacity and metabolic resilience in *KRAS*-mutant cancers 36. The concurrent induction of mitochondrial markers (*HSP60*, *SOD2*) and lipid metabolism regulators (*FABP-1*, *PON-2*) indicates NB-induced perturbation of the lipid-redox balance, further supporting mitochondrial involvement in the adaptive response.

In sum, this study reveals that NB-cell interactions extend beyond simple internalization and are tightly coupled to the genetic and metabolic state of the target cell. We propose that the lipid-mimetic properties of NBs enable direct interactions with cellular and organellar membranes, thereby perturbing lipid-sensing and redox pathways which can have different consequences in different cell types. In a PDAC wild-type cell, this may result in a localized oxidative and inflammatory defence, whereas in a PDAC KRAS-mutant cell, metabolic rewiring may enable a more integrated cytoprotective adaptation, highlighting the dual role of NBs as both a nanocontrast agent and a biological stimulus that engages intrinsic stress and signalling networks. Also, the observed cell-type-specific intracellular localization and retention of TR-NBs provide a mechanistic basis for spatially heterogeneous contrast generation in tumor imaging, supporting their further evaluation in advanced imaging models.

## Conclusion

Collectively, our findings define a previously underappreciated dimension of NB-cell interactions. Non-targeted, perfluorocarbon-filled NBs are internalized via rapid, cell-type-specific endocytic pathways that are independent of serum proteins, highlighting an intrinsic cellular uptake mechanisms. Beyond their conventional roles as drug-delivery carriers and contrast agents, NBs function as active nanoscale probes that report on cell-type-specific endocytic routing and trafficking kinetics, including time- and temperature-dependent intracellular behavior and differential stress responses between cell lines. Future *in vivo* studies integrating high-resolution imaging and US modulation will be essential to translate these mechanistic insights into precision nanotheranostic strategies.

## Supplementary Material

Supplementary figures and tables.

## Figures and Tables

**Figure 1:**
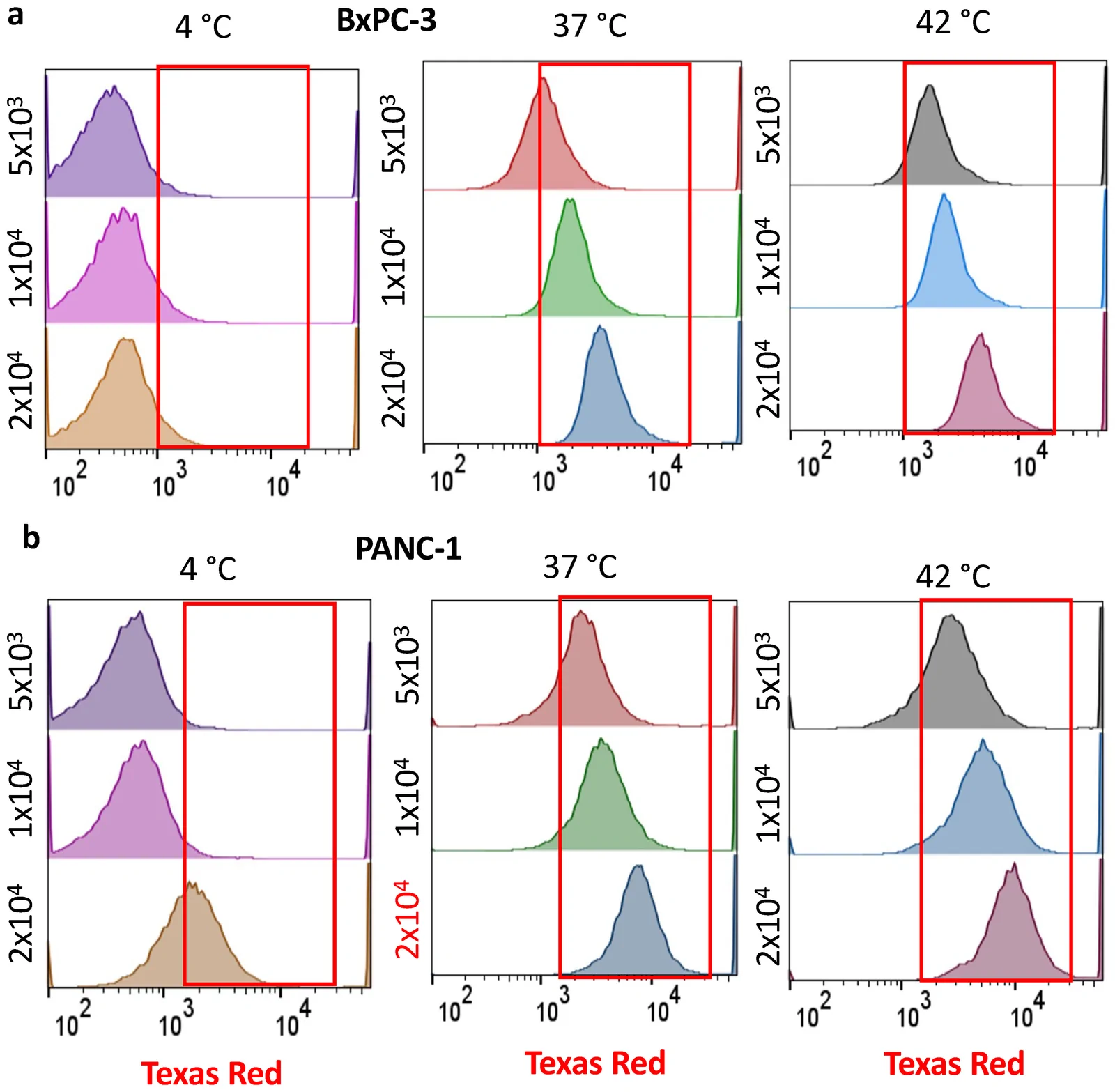
** Selected NB to Cell Ratio.** Flow cytometric analysis was performed to evaluate the internalization of TR-labeled nanobubbles (TR-NBs) in BxPC-3 and PANC-1 cells using 2 × 10^4^, 1 × 10⁴, and 5 × 10³ NBs per cell at 4 °C (to assess non-specific binding) and 37 °C (to assess internalization) (a,b). At 4 °C, fluorescence intensities remained low and comparable across all NB numbers, indicating minimal passive association of TR-NBs with the cell surface. In contrast, at 37 °C, both cell lines showed a marked increase in fluorescence intensity, as evidenced by a rightward shift in the peak, confirming temperature-dependent internalization. Among tested conditions, 1 × 10⁴ NBs yielded a strong and consistent internalization signal at 37 °C.

**Figure 2:**
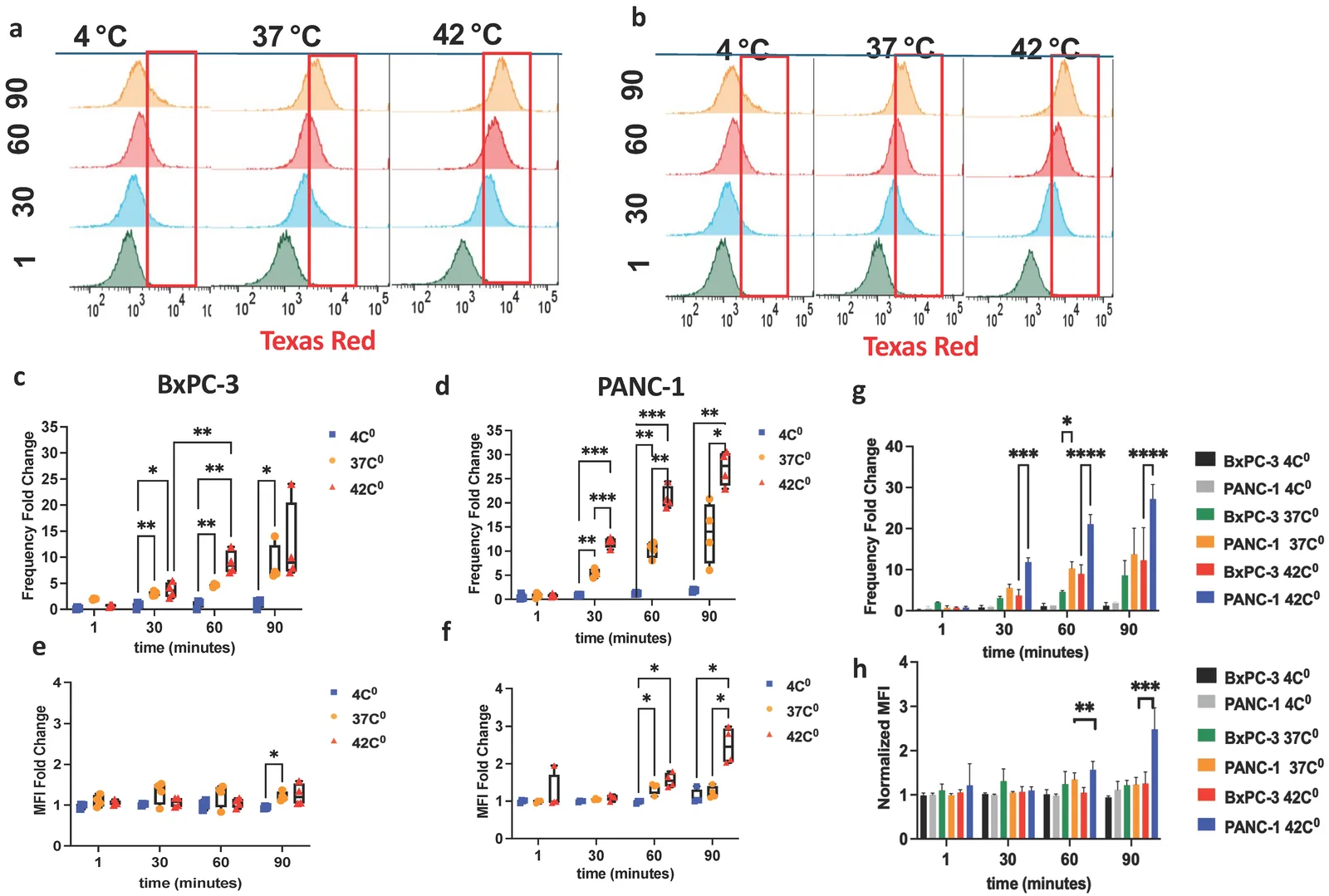
** Time- and temperature-mediated NB internalization.** Flow cytometric analysis of TR-NB internalization BxPC-3 (a) and PANC-1 cells (b) at 4°C, 37°C, and 42°C, over 1, 30, 60 and 90 minutes. Representative histograms show fluorescence intensity shifts corresponding to NB internalization under each condition. BxPC-3 cells exhibited a progressive, temperature-dependent increase in internalization, with significantly higher uptake at 37 °C and 42 °C compared with 4 °C (p < 0.05 to p < 0.01) (c). PANC-1 cells exhibited a time- and temperature-dependent increase in TR-NB uptake, with highest values at later time points, particularly at 42°C (d). Direct comparison with BxPC-3 cells is shown in panels g and h. (d). MFI remained relatively stable across 37 °C in BxPC-3 but increased at 42 °C (e), whereas MFI showed a significant increase in PANC-1 across temperatures and time points (f). Combined frequency and MFI showing the comparison between two cell types across the time points and temperatures (g,h). Data represent mean ± SD of three independent experiments. Statistical significance was determined using two-way ANOVA with post hoc testing (* p < 0.05, ** p < 0.01, **** p < 0.0001).

**Figure 3:**
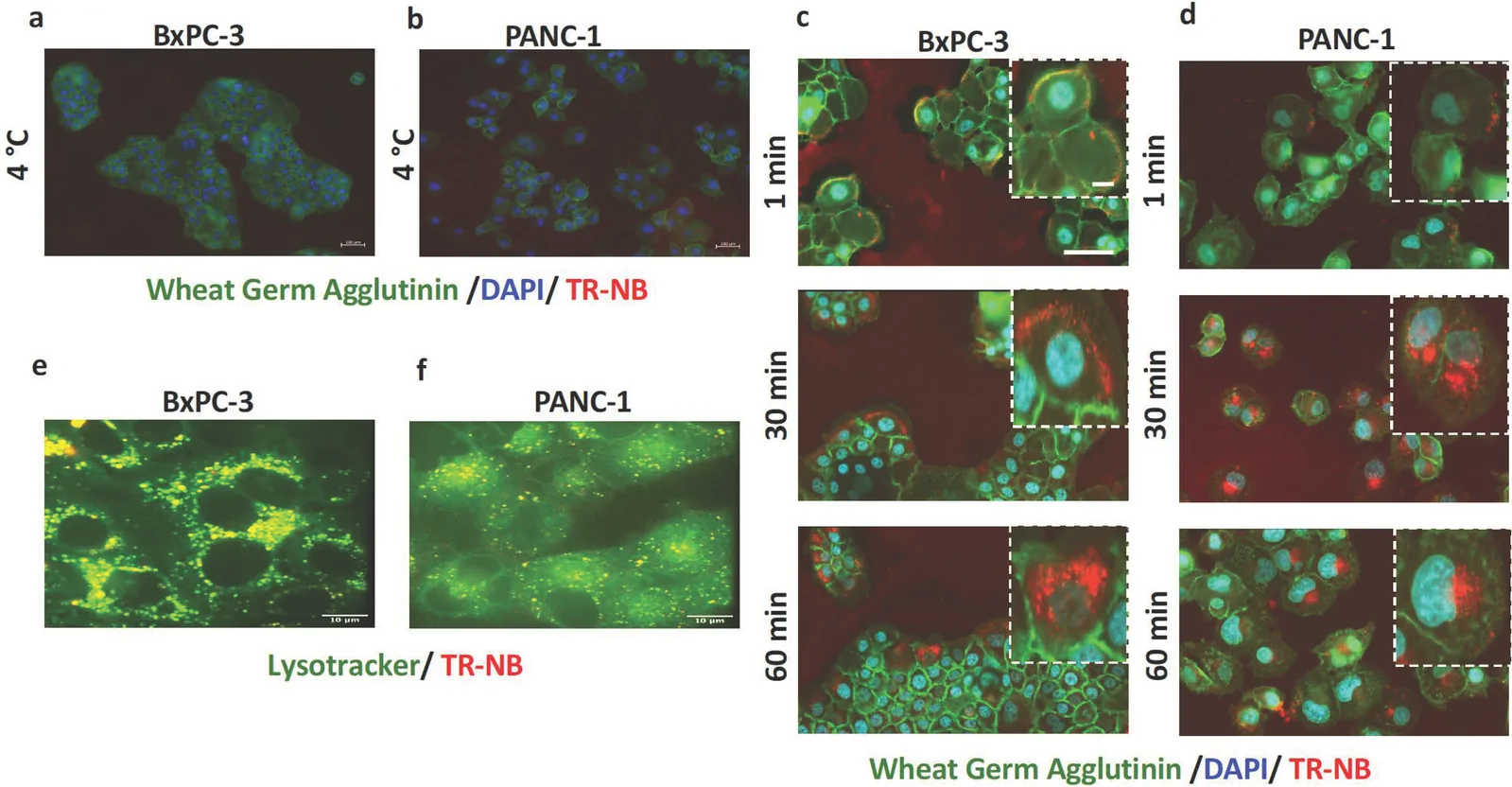
**Microscopic visualization of NB internalization.** Wide-field fluorescence images of BxPC-3 (a) and PANC-1 (b) cells incubated with TR-NBs at 4 °C for 60 minutes. Almost no red fluorescence is observed on the cells under these conditions. Nuclei are stained blue (DAPI), cell membranes green (WGA), and TR-NBs red (Texas Red). Scale bar, 100 μm. Time-course images of NB internalization at 37 °C in BxPC-3 (c) and PANC-1 (d) cells at 1, 30, and 60 minutes. Insets show magnified single-cell regions (dashed outlines) highlighting the progressive change in NB localization. At 1 minute, NBs are predominantly membrane-associated; by 30 minutes, they are distributed throughout the cytoplasm; and by 60 minutes, they accumulate in the perinuclear region, particularly in PANC-1 cells (d). Scale bars: 50 μm (wide field), 10 μm (insets). Lysosomal compartments are labeled with Lysotracker Green (green), TR-NBs are shown in red, and nuclei are counterstained with DAPI (blue). BxPC-3 cells display strong Lysotracker staining with a perinuclear distribution (e), whereas PANC-1 cells exhibit weak Lysotracker signal localized to discrete perinuclear regions (f). Scale bar: 10 µm.

**Figure 4:**
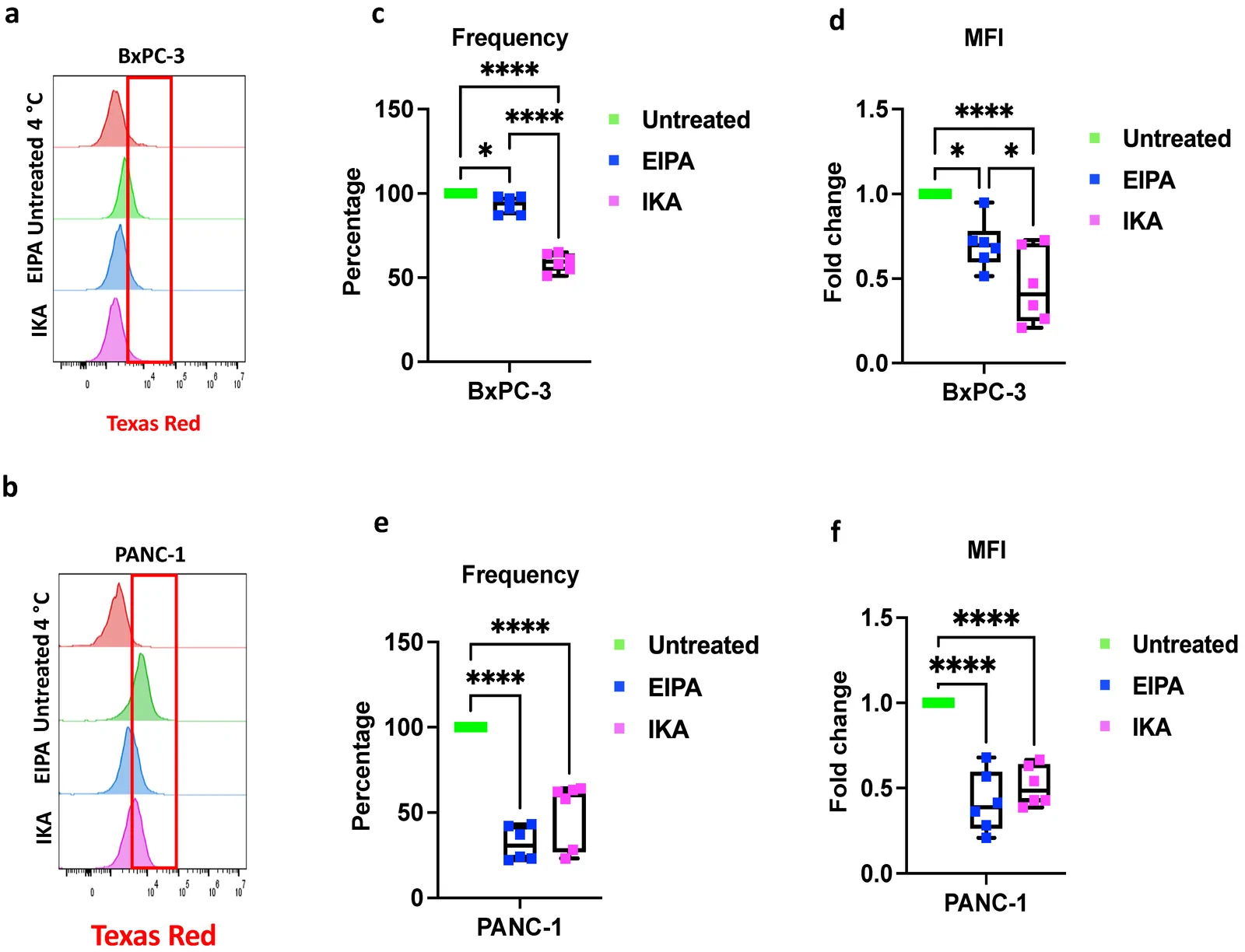
** Inhibition of NB internalization via CME and macropinocytosis-mediated pathways.** BxPC-3 (a) and PANC-1 cells (b) were pre-treated with ikarugamycin (IKA; clathrin-mediated endocytosis inhibitor) or EIPA (macropinocytosis inhibitor) prior to exposure to TR-labeled nanobubbles (TR-NBs) at 37 °C. In BxPC-3 cells, IKA reduced NB-positive cells by 41% (c) and MFI by 55% (d), whereas EIPA caused smaller decreases 7% (c) and 30% (d), indicating a predominant role for clathrin-mediated uptake. In PANC-1, EIPA reduced NB-positive cells by 69.2% (e) and MFI by 58% (f), compared with 49.6% (e) and 48% (f) reductions following IKA, indicating macropinocytosis as the dominant internalization mechanism. Data are shown as mean ± SD (n = 3). *p < 0.05, ****p < 0.0001 (one-way ANOVA with Tukey's post hoc test).

**Figure 5:**
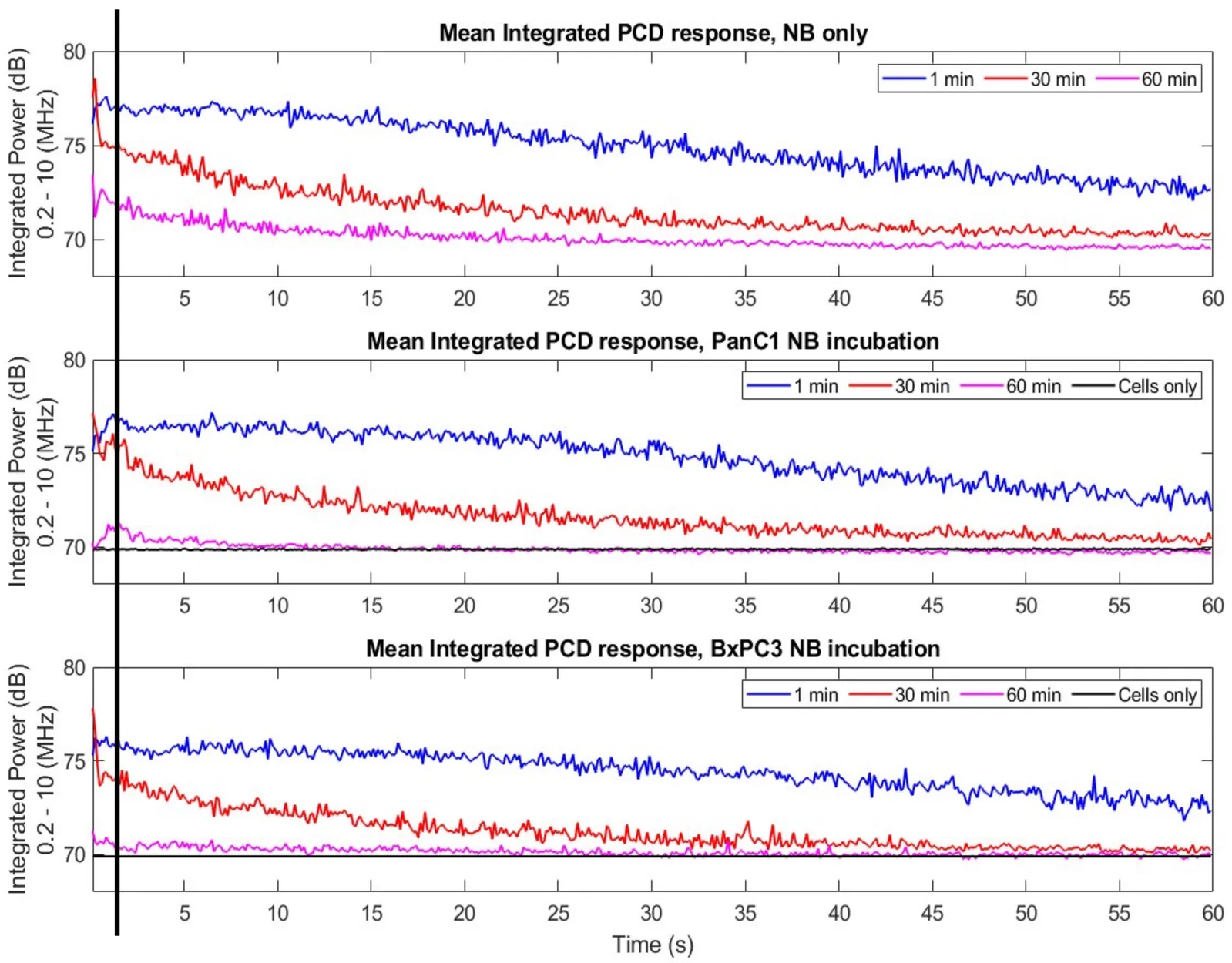
** US response.** Normalized mean integrated power spectra obtained via Passive Cavitation Detection (PCD) (n = 8) showing the acoustic activity of PGG-NBs under three conditions: NBs in media only (top), NBs incubated with PANC-1 cells (middle), and NBs incubated with BxPC-3 cells (bottom). Samples were incubated at 37 °C for 1 min (blue), 30 min (red), or 60 min (magenta) prior to US exposure. Cell-only controls (black) are shown for reference in the middle and bottom panels. Integrated power values were normalized to the RF signal of cell-only controls. Initial higher signal for PANC-1 is indicated by black vertical line. The progressive decline in integrated power over time indicates a loss of acoustic activity, consistent with NB internalization, cellular interaction, or collapse.

**Figure 6:**
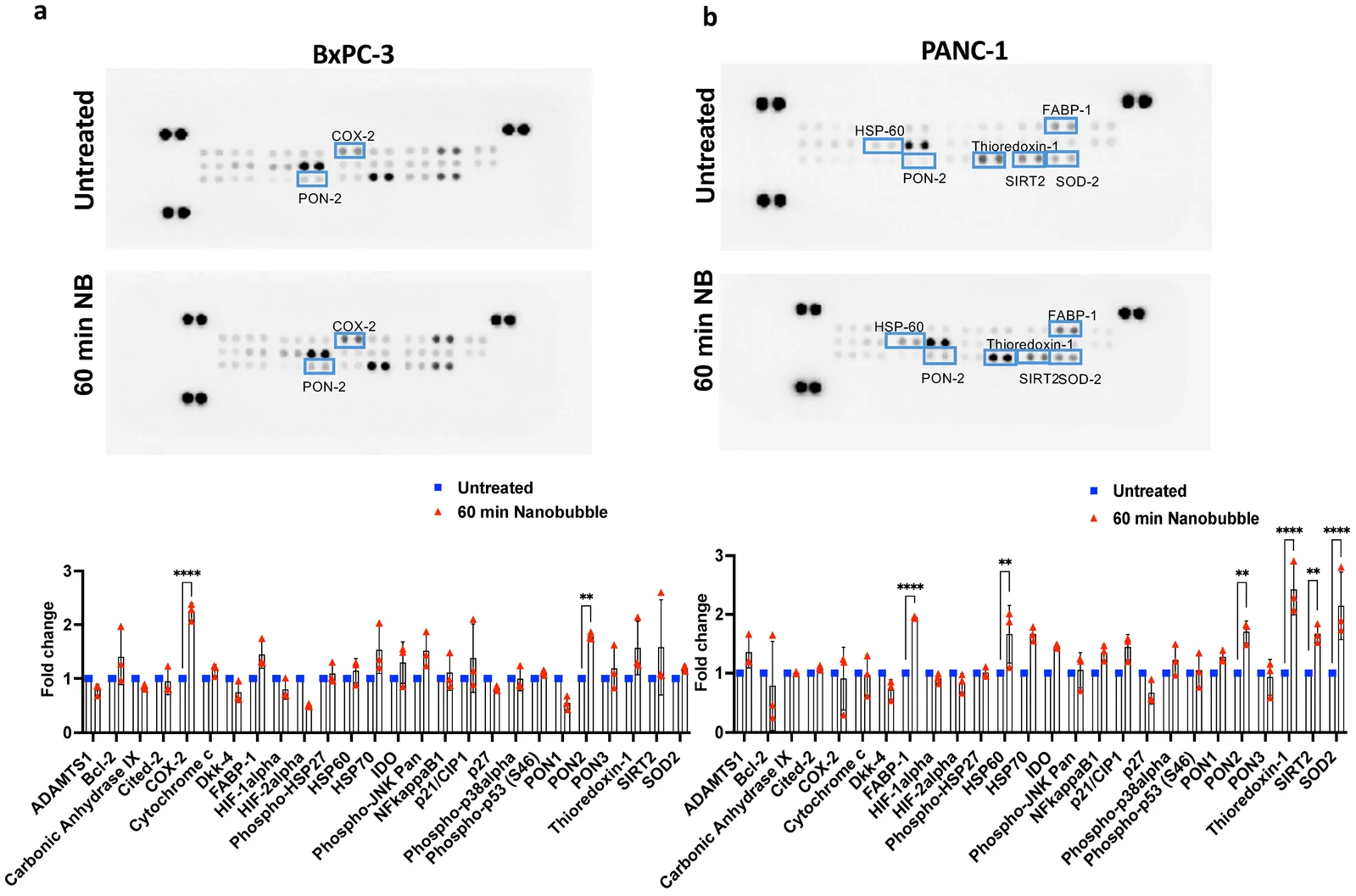
** NB exposure induces distinct cellular stress response signatures.** (a) BxPC-3 and (b) PANC-1 cells were treated with PGG-NBs for 60 minutes at 37 °C, and protein lysates were analyzed using a human cell stress protein array. Representative blots (top panels) show protein expression profiles in untreated versus NB-treated cells, with key upregulated proteins annotated. Quantification of relative protein expression (bottom panels) is shown as fold change compared with untreated controls. In BxPC-3 cells, NB treatment primarily increased COX-2 and PON-2 expression, indicating activation of oxidative and inflammatory stress pathways (a). In contrast, PANC-1 cells displayed a broader stress response, with more than 2-fold upregulation of HSP60, Thioredoxin-1, FABP-1, SOD2, and SIRT2, consistent with a more extensive metabolic and proteostatic adaptation (b). Data represent mean ± SD (n = 3). Statistical analysis was performed using two-way ANOVA followed by Sidak's multiple comparison test. **p < 0.01, ****p < 0.0001.

## Abbreviations

CME: clathrin-mediated endocytosis
MBs: microbubbles
NBs: nanobubbles
PCD: passive cavitation detection
PDAC: pancreatic ductal adenocarcinoma
US: ultrasound
TR: Texas Red
