# Supplementary material for: Distinct Internalization, Acoustic Behavior and Stress Responses to Nanobubbles in Pancreatic Cancer Cells
Source: Nanotheranostics. 2026 Sep 7;10:210–23. doi: 10.7150/ntno.130552 (PMC13619351; doi:10.7150/ntno.130552)
Supplement: Supplementary file 1 [file ntnov10p0210s1.pdf]

Supplemental Table 1: Physicochemical characteristics of PGG-NBs measured by ARMM.

Number	Positive Buoyancy (%)	Avg Diameter (µm)	Concentration (nb/ml) (x10^8)
1	91.3	0.252	6.91
2	88.0	0.264	7.69
3	90.1	0.276	7.62
4	84.7	0.294	8.88
5	100	0.256	7.35
**Average**	**90.8**	**0.26**	**7.69**

Supplemental Table 2: Physicochemical characteristics of TR-NBs measured by ARMM.

Number	Positive Buoyancy (%)	Avg Diameter (µm)	Concentration (nb/ml) (x10^8)
1	87.7	0.264	2.79
2	90.1	0.276	4.46
3	91	0.299	5.11
4	87.1	0.23	3.91
5	85.6	0.303	6.02
6	90.3	0.312	6.61
7	93.9	0.323	8.08
8	76.5	0.291	2.52
9	88.3	0.318	5.7
10	86.7	0.306	3.58
11	85.6	0.316	5.39
12	86.5	0.31	5.51
13	82.1	0.227	4.12
14	89.6	0.314	4.73
15	70.5	0.29	1.21
16	91.1	0.353	8.48
17	86.1	0.268	3.05
**Average**	**86.4**	**0.29**	**4.78**

Supplemental Figure Legends:

Figure S1: Flow cytometry gating strategy for TR-NB uptake analysis in pancreatic cells.

(a) Sequential gating strategy used to identify viable single pancreatic cells for uptake analysis. Total events were first gated based on forward scatter area (FSC-A) versus side scatter area (SSC-A) to select the main pancreatic cell population and exclude debris. Doublets were removed using FSC-A versus FSC-H, followed by singlet refinement using SSC-H versus SSC-W. Live cells were then selected by excluding DAPI-positive events. The resulting live singlet population was used for downstream quantification of TR-NB uptake and for histogram overlay visualization. Texas Red fluorescence-positive events within the predefined uptake gate were quantified for statistical analysis.

(b) Representative gating strategy used to quantify time-dependent TR-NB uptake at 4°C and 37°C. A DAPI single-stain control was used at each time point to define the Texas Red-positive uptake gate (NB+). Representative dot plots show the percentage of nanobubble-positive cells at 0, 30, 60, and 90 min under both temperature conditions. Uptake increased progressively over time and was markedly enhanced at 37°C compared with 4°C, consistent with temperature-dependent cellular internalization.

Figure S2. Dose-dependent effect of ikarugamycin (IKA) on the viability of BxPC-3 pancreatic cancer cells. BxPC-3 cells were treated with increasing concentrations of IKA, and cell viability was determined using the Vi-CELL trypan blue exclusion assay (a). A concentration of 1.5 µM IKA was selected for subsequent studies as it effectively inhibited CME (b) while maintaining high cell viability.

Figure S3. **Effect of EIPA on macropinocytosis in PANC-1 and BxPC-3 pancreatic cancer cells.** Cells were incubated with FITC-labeled 70 kDa dextran (green) to assess macropinocytic uptake, with or without the Na^+/H^+ exchanger inhibitor EIPA. Nuclei were counterstained with DAPI (blue). PANC-1 cells display more substantial dextran uptake than BxPC-3 cells (a,b) indicating higher basal macropinocytic activity. EIPA treatment markedly reduces dextran internalization in both cell lines, confirming inhibition of macropinocytosis. Scale bar: 50 µm.

Figure S4. Effect of endocytic pathway inhibitors on the viability of PANC-1 and BxPC-3 pancreatic cancer cells. Cells were treated with ikarugamycin (IKA, 1.5 µM for BxPC-3, 4 µM for PANC-1) or EIPA (50 µM), and viability was assessed by flow cytometry using efluor 780 (e780) fixable viability dye. Both inhibitors showed minimal cytotoxicity at these concentrations (a,b). Data represent mean ± SD from three independent experiments.

Figure S5. Effect of US exposure on the viability of PANC-1 and BxPC-3 pancreatic cancer cells. Cells were incubated with nanobubbles and exposed to US. Cell viability was determined using the Vi-CELL trypan blue exclusion assay. Both cell lines maintained high viability following US treatment, confirming that the acoustic parameters used did not induce significant cytotoxicity (a,b). Data represent mean ± SD from three independent experiments.

Figure S6. Time-dependent turbidity of NB suspensions with and without cells before and after US exposure. Representative photographs from a single experiment show untreated (a) and US-treated (b) suspensions containing cells alone, NBs alone, or cells + NBs after incubation with BxPC-3 or PANC-1 cells for the indicated times. NB-only suspensions gradually became less turbid over time and appeared visibly clearer following US exposure. Optical density at 600 nm (OD600) was measured before and after US treatment for suspensions containing cells alone, NBs alone, or cells + NBs following incubation at 37°C for 1, 30, or 60 min with BxPC-3 (c) or PANC-1 (d) cells. At 1 min, NB-containing groups showed a pronounced reduction in OD after US exposure, consistent with disruption of suspended acoustically responsive NBs. At later time points (30 and 60 min), the magnitude of OD reduction progressively decreased. By 60 min, OD values of the cell + NB groups approached those of cell-only controls, suggesting that fewer extracellular NBs remained in the medium.

Figure S7. Human cell stress proteome array layout used for targeted protein analysis.

Schematic of the Human Cell Stress Array coordinates and corresponding analytes. Each antibody pair is spotted in duplicate according to the grid shown. The array includes markers for stress proteins along with appropriate reference and negative control spots. Coordinates correspond to analytes listed in the accompanying table.

Supplemental Figure 1a

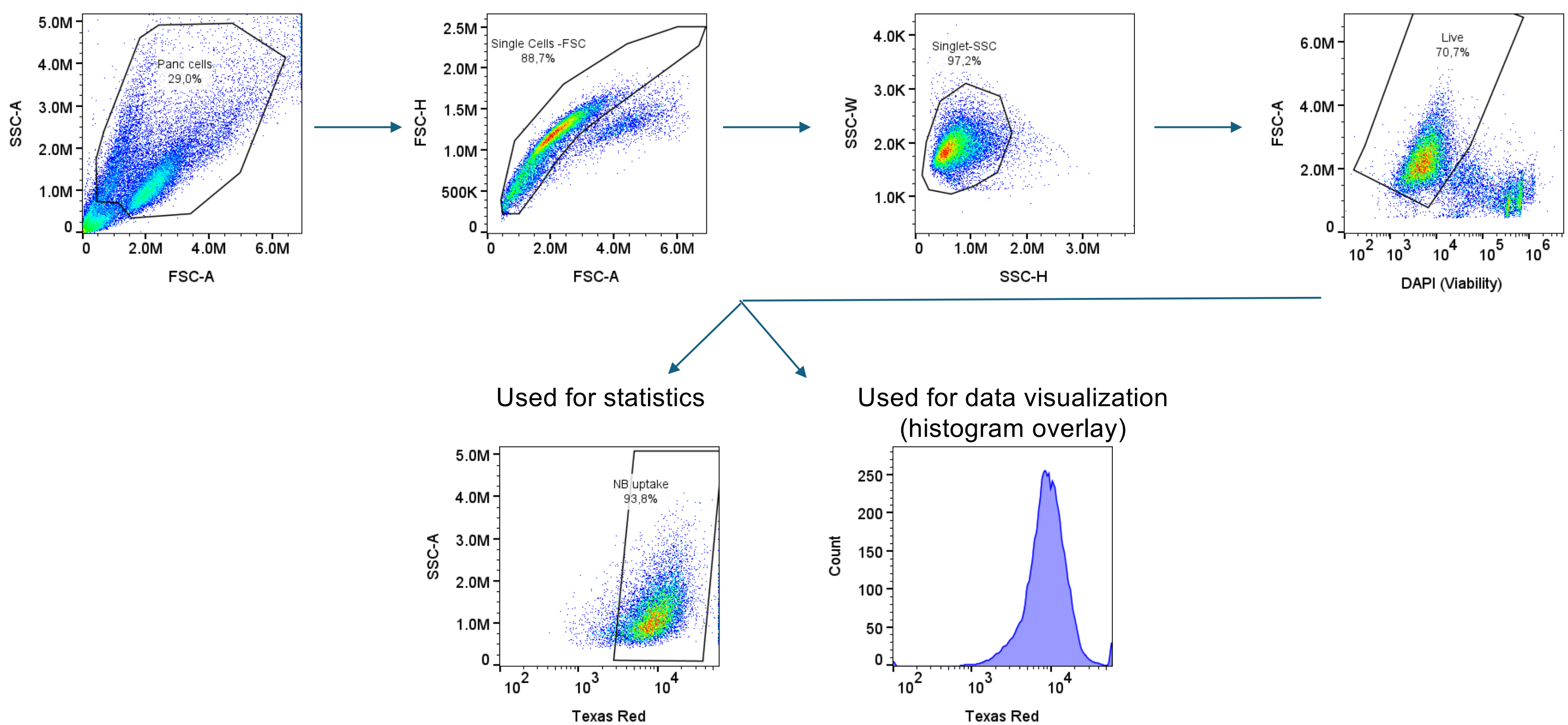

Supplemental Figure 1b

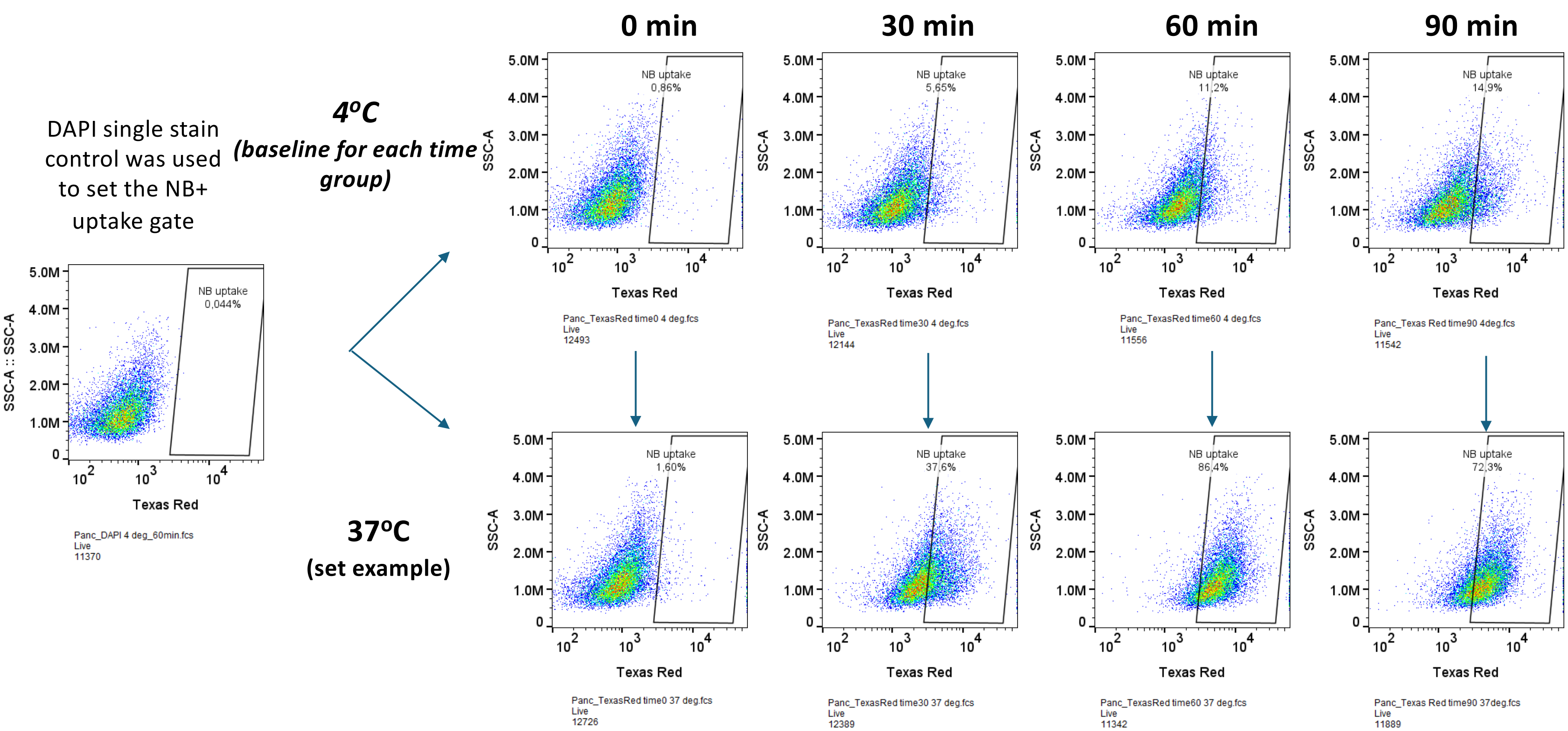

Supplemental Figure 2

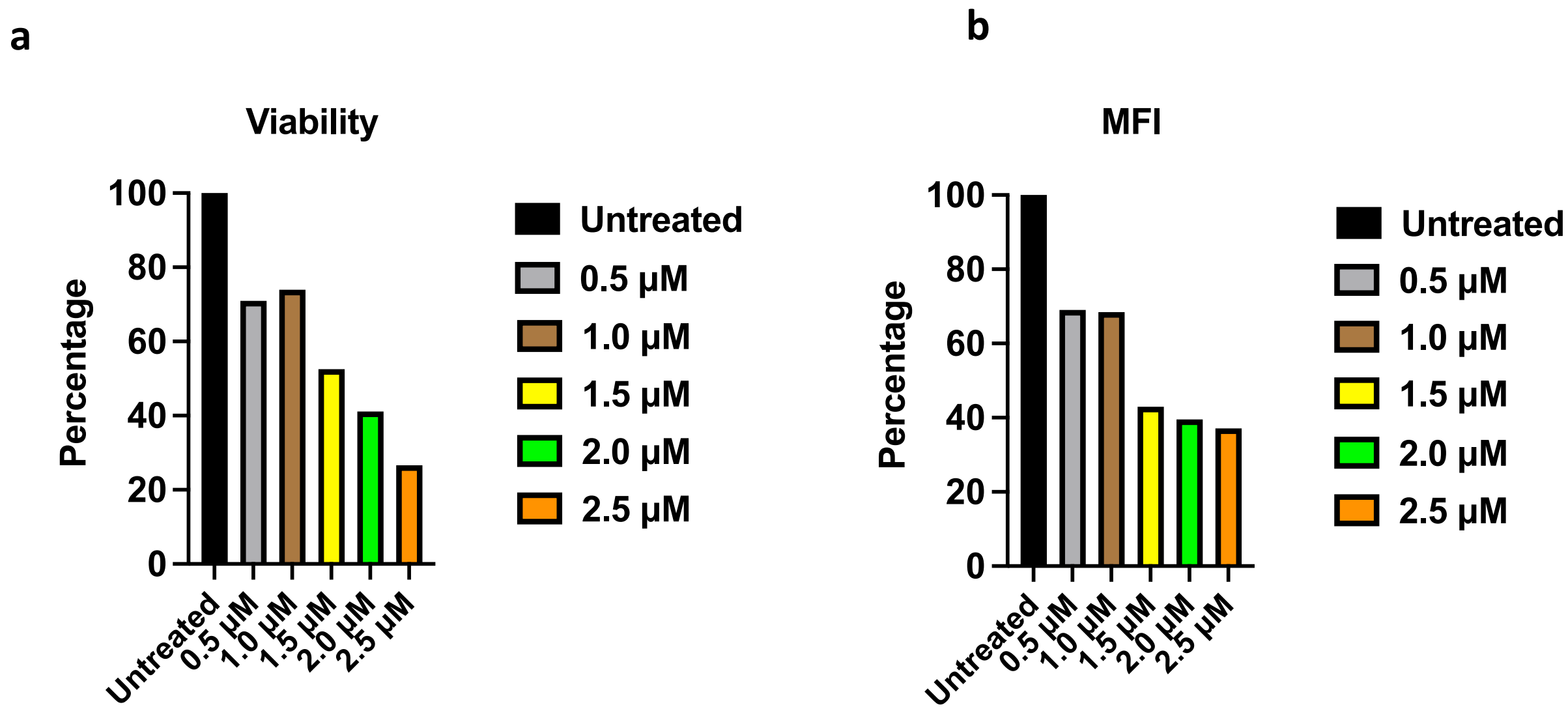

Supplemental Figure 3

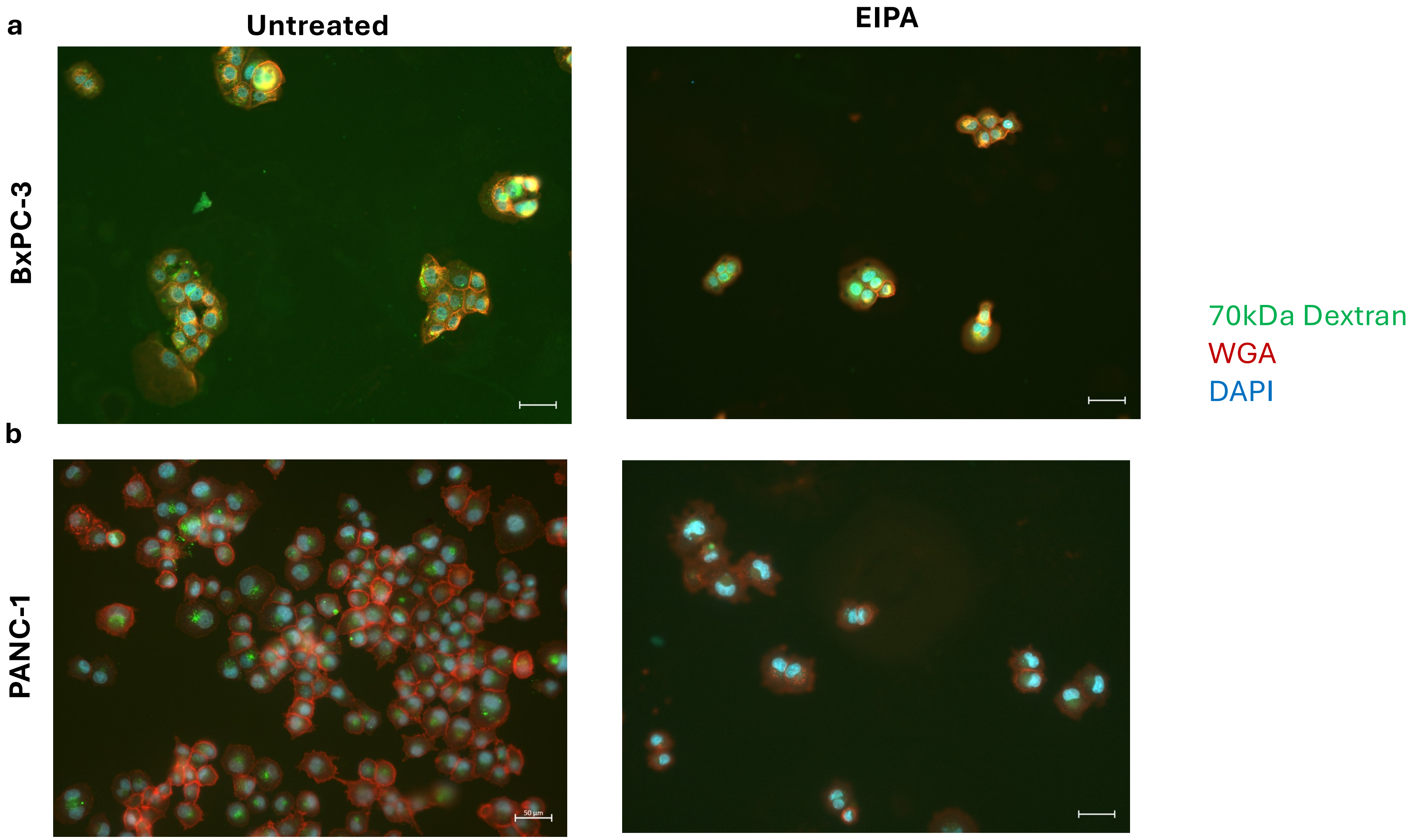

Supplemental Figure 4

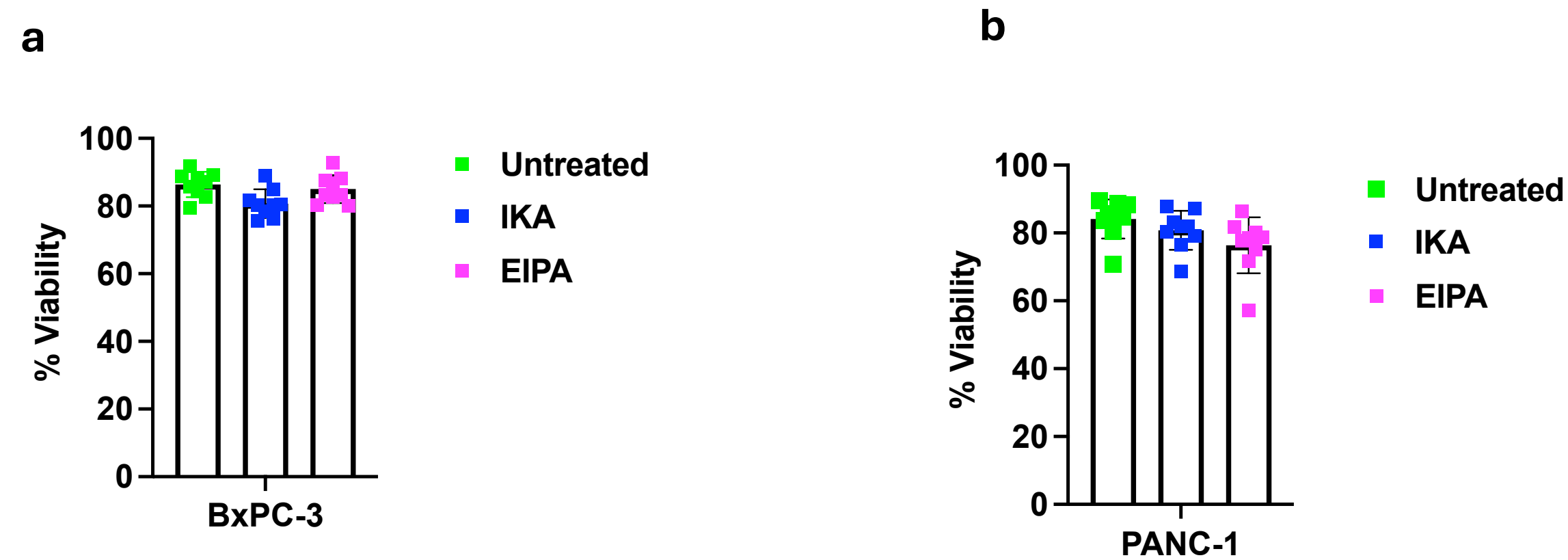

Supplemental Figure 5

a

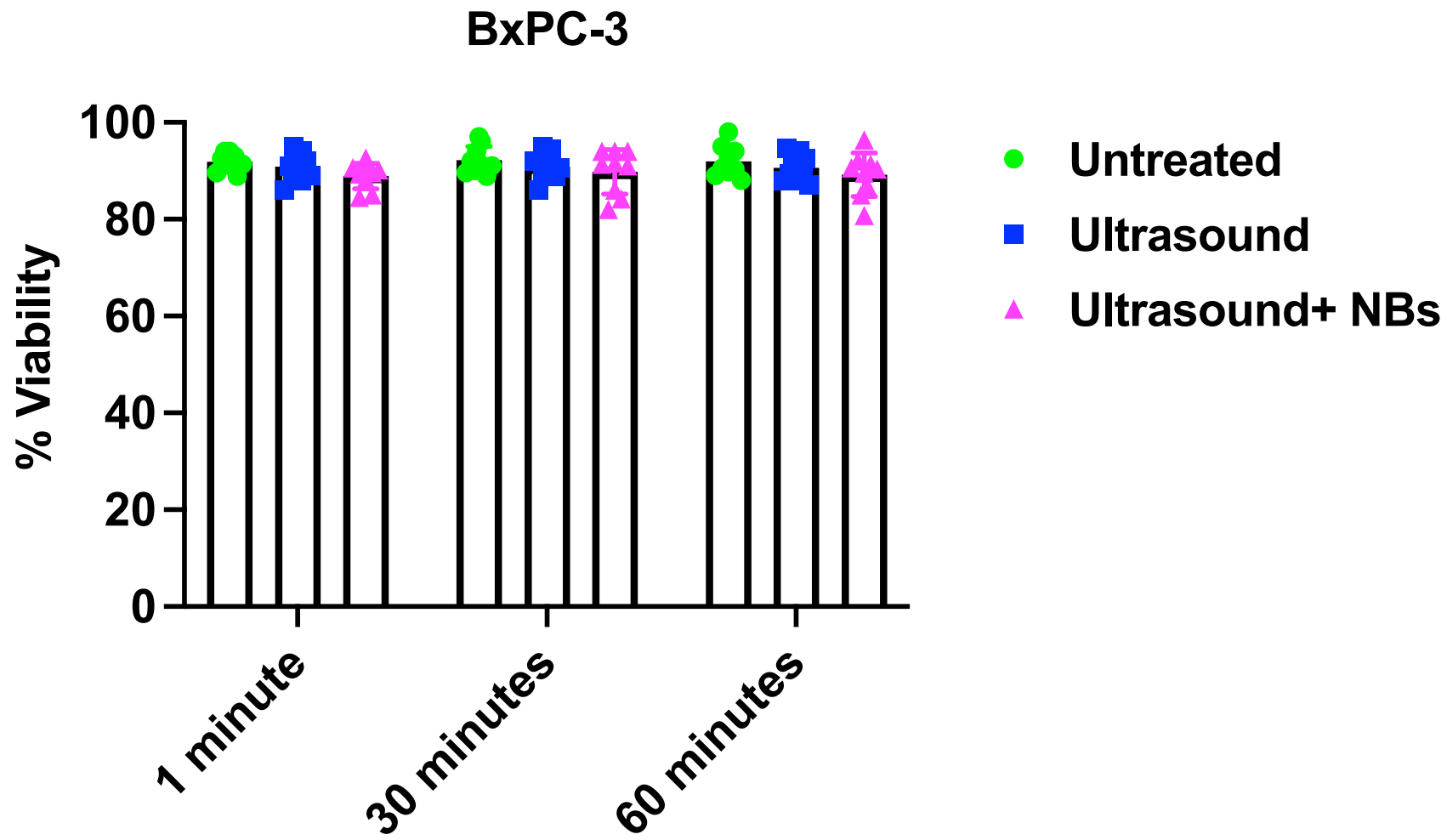

b

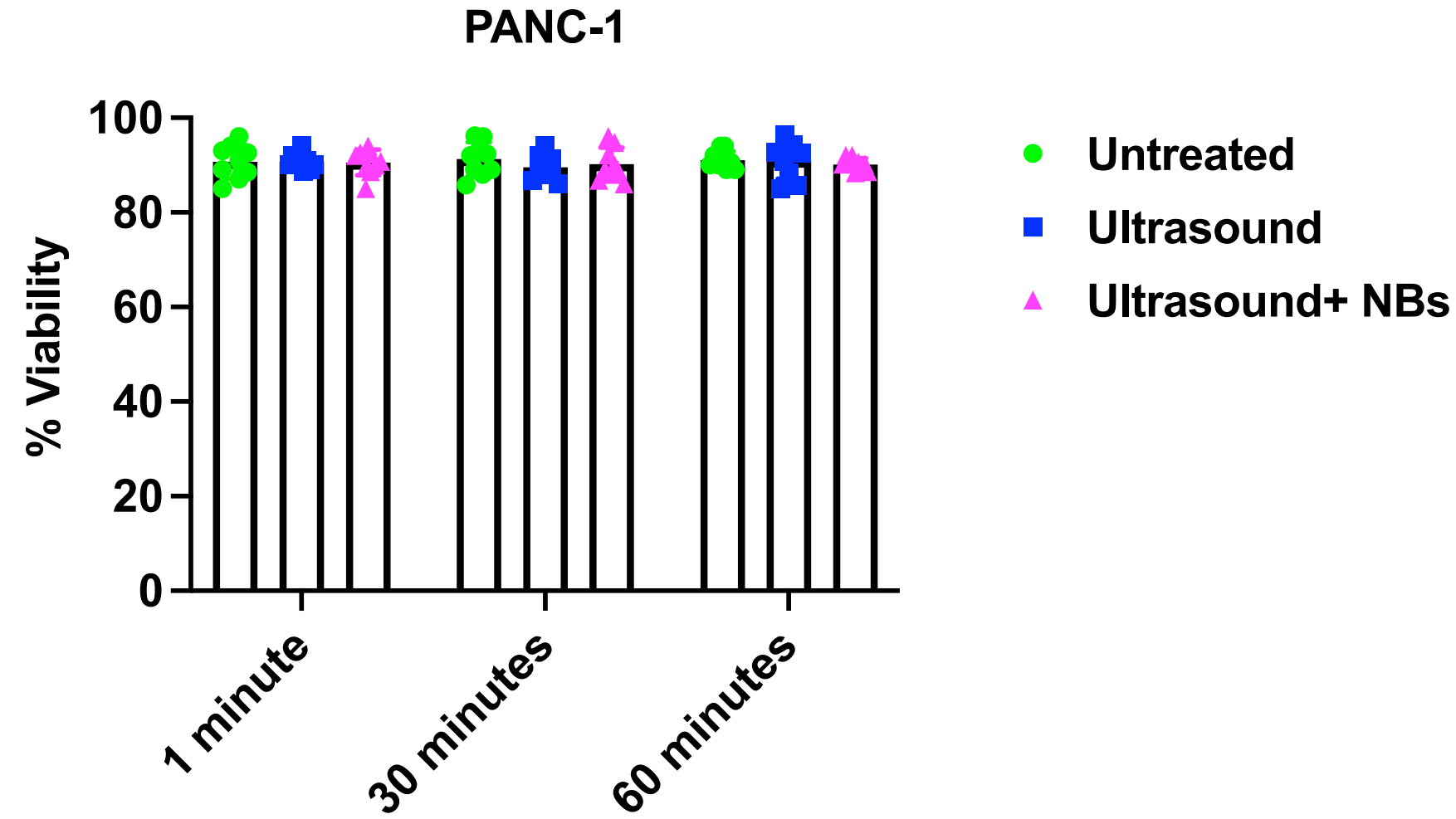

Supplemental Figure 6

a

Untreated

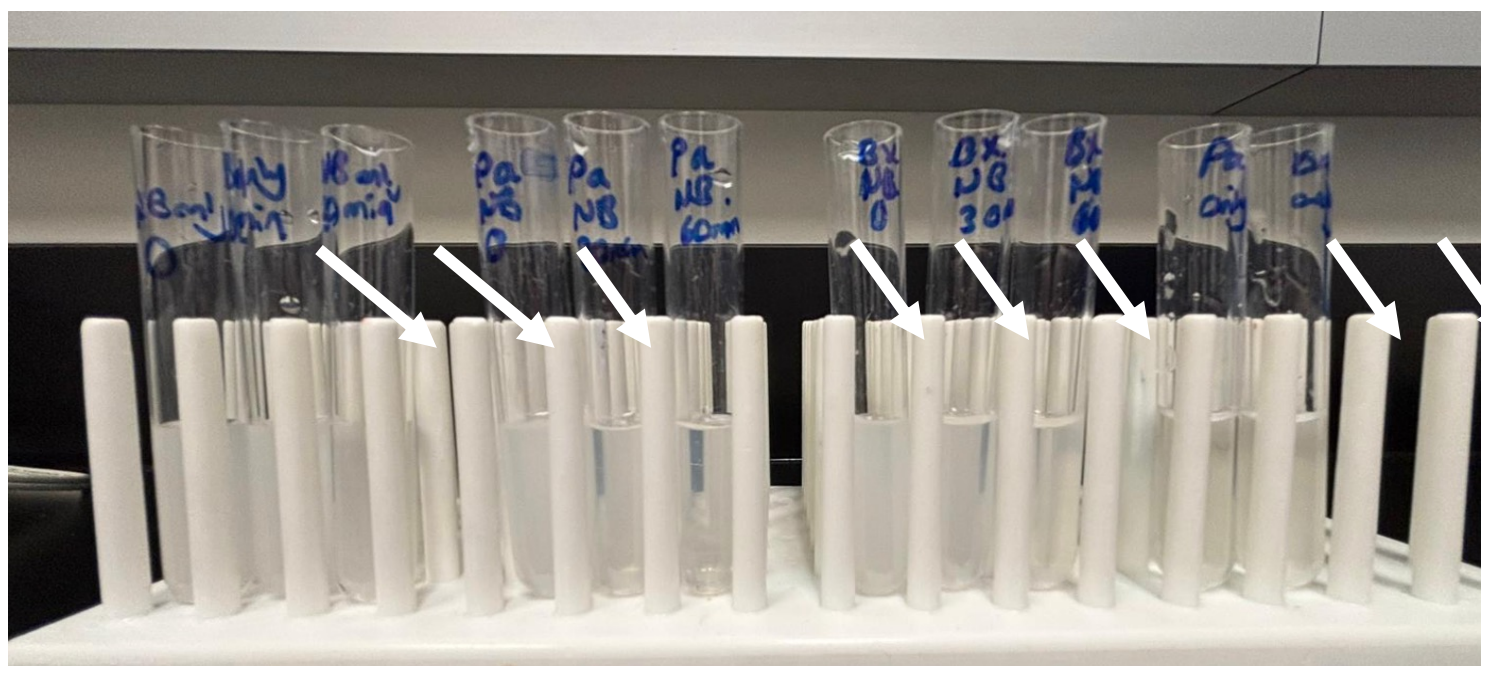

b

Ultrasound Treated

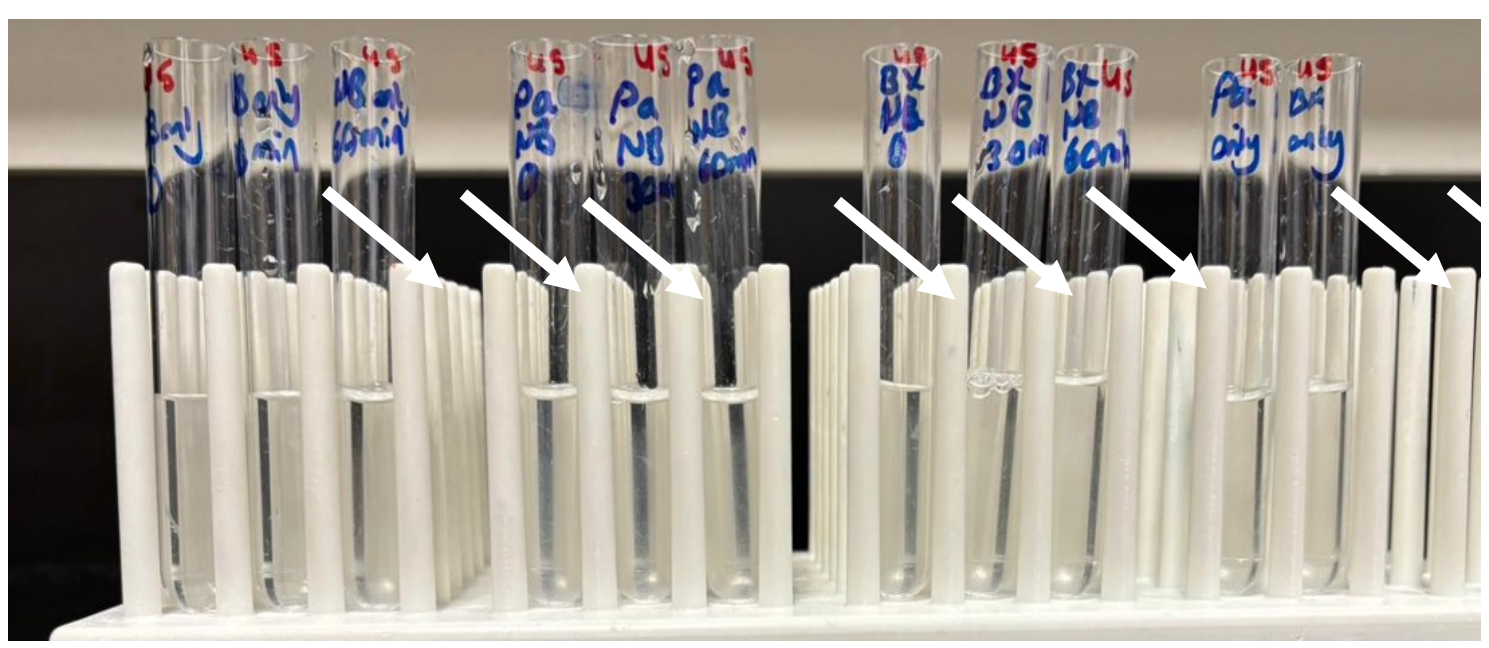

c

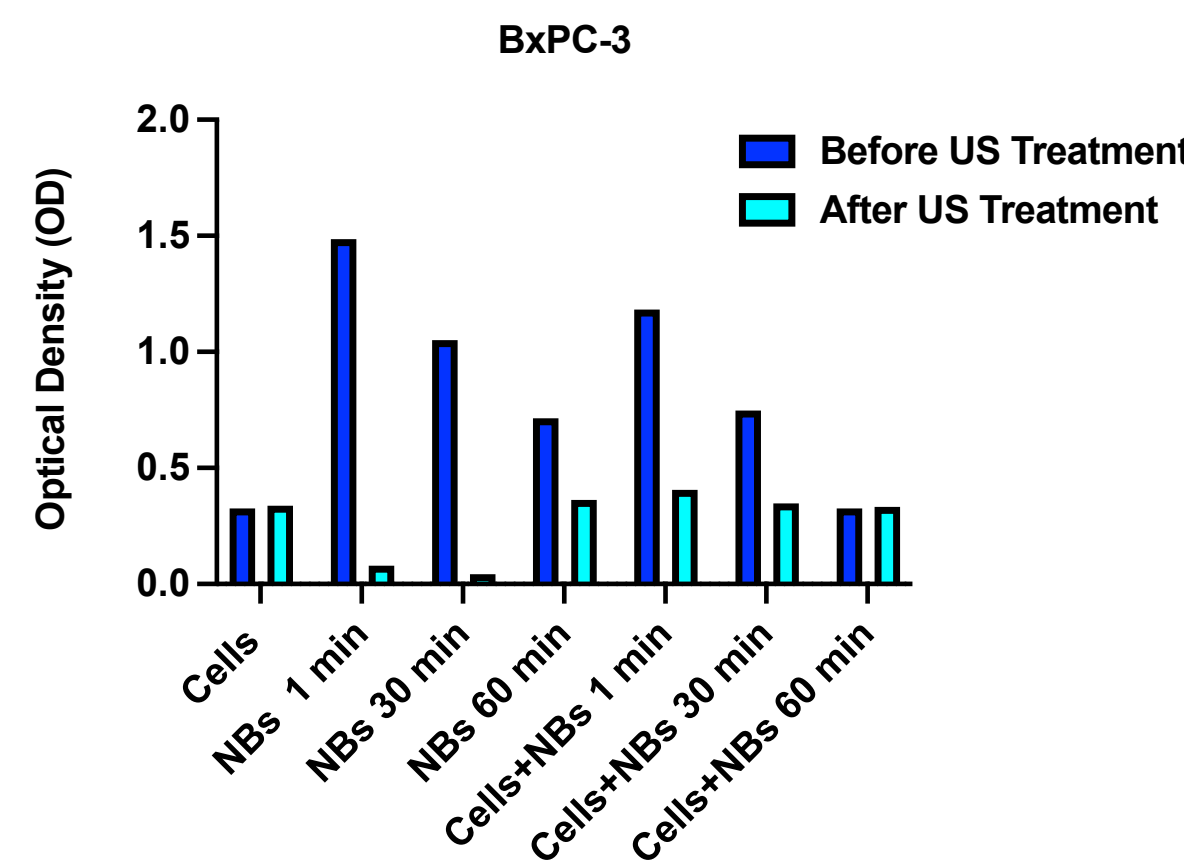

d

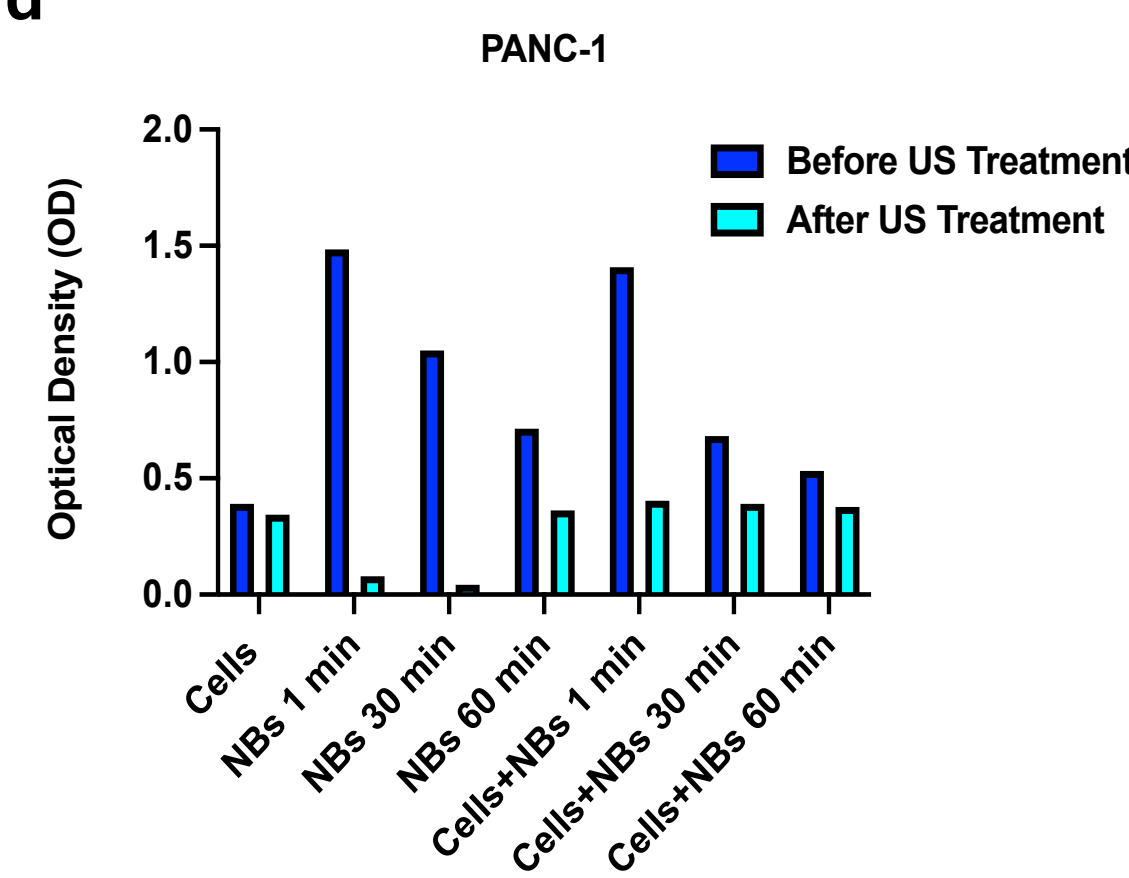

Supplemental Figure 7

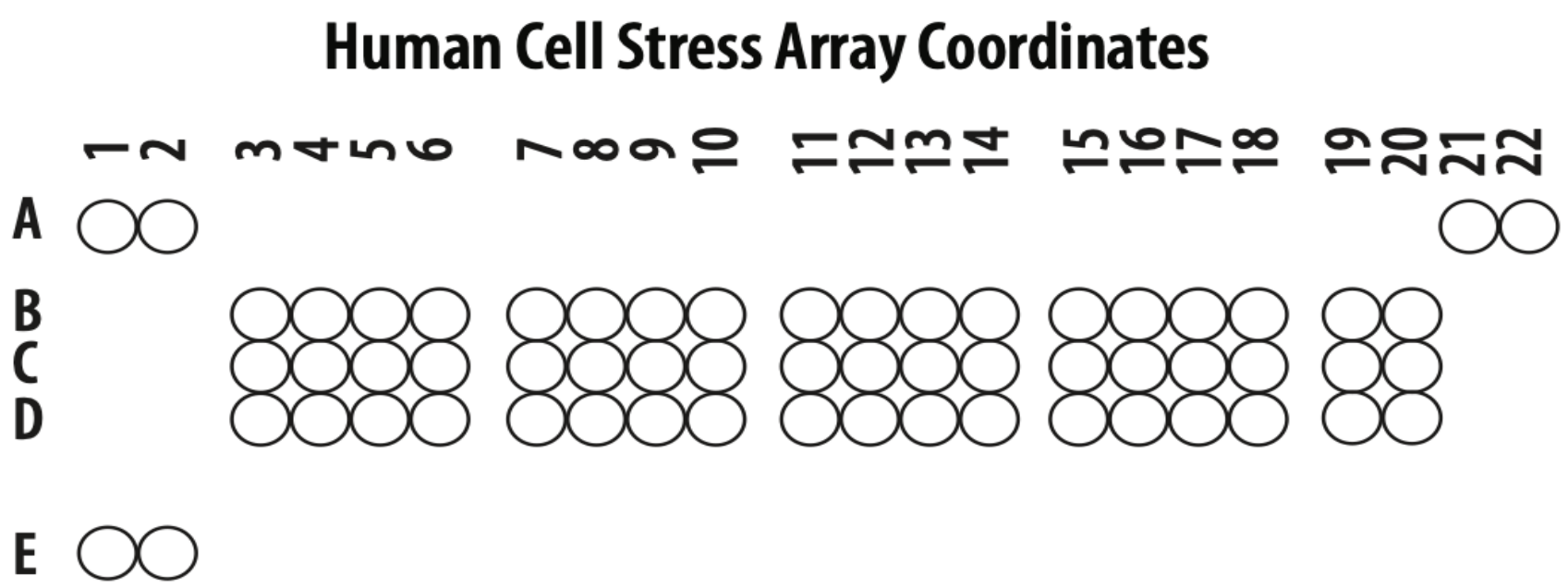

Coordinate	Analyte/Control	Alternate Nomenclature
A1, A2	Reference Spots	——
A21, A22	Reference Spots	——
B3, B4	ADAMTS1	——
B5, B6	Bcl-2	——
B7, B8	Carbonic Anhydrase IX	CA9
B9, B10	Cited-2	——
B11, B12	COX-2	——
B13, B14	Cytochrome c	——
B15, B16	Dkk-4	——
B17, B18	FABP-1	L-FABP
B19, B20	HIF-1α	——
C3, C4	HIF-2α	EPAS1
C5, C6	Phospho-HSP27 (S78/S82)	——
C7, C8	HSP60	——
C9, C10	HSP70	——
C11, C12	IDO	Indoleamine 2,3-dioxygenase
C13, C14	Phospho-JNK Pan (T183/Y185)	——
C15, C16	NFκB1	——
C17, C18	p21/CIP1	CDNK1A
C19, C20	p27	Kip1
D3, D4	Phospho-p38α (T180/Y182)	——
D5, D6	Phospho-p53 (S46)	——
D7, D8	PON1	——
D9, D10	PON2	——
D11, D12	PON3	——
D13, D14	Thioredoxin-1	——
D15, D16	SIRT2	Sirtuin 2
D17, D18	SOD2	Mn-SOD
D19, D20	Negative Control	Control (-)
E1, E2	Reference Spots	——
